# Solution-plasma-derived Fe–N–C/NiFe-LDH composite for bifunctional oxygen electrocatalysis: effects of surface modification and LDH growth

**DOI:** 10.1039/d6ra07919a

**Published:** 2026-10-16

**Authors:** Kentaro Ogihara, Kouki Yamamoto, Hayato Takada, Takahiro Ishizaki

**Affiliations:** a Materials Science and Engineering, Graduate School of Engineering and Science, Shibaura Institute of Technology Toyosu, Koto-Ku Tokyo 135-8548 Japan; b College of Engineering, Shibaura Institute of Technology Toyosu, Koto-Ku Tokyo 135-8548 Japan ishizaki@shibaura-it.ac.jp

## Abstract

Herein, a noble-metal-free bifunctional electrocatalyst was synthesized by growing NiFe layered double hydroxide (NiFe-LDH) nanosheets *via* a hydrothermal method on a solution-plasma-derived Fe–N–C substrate for rechargeable air-battery applications. Nitric acid modification increased the amount of oxygen-containing surface groups, including O–C=O species, which can serve as anchoring sites for subsequent LDH nucleation. However, acid treatment also decreased the surface N content and the N 1s component assigned to Fe–N coordination, resulting in a trade-off between surface functionalization and preservation of ORR-active structures. SEM/EDS and XRD supported the formation of a NiFe-LDH phase on the Fe–N–C-based support, while the approximately +0.3 eV Ni 2p binding-energy shift observed by XPS indicated a change in the local chemical/electronic environment upon composite formation. The present data, however, do not directly establish an atomically intimate heterointerface or the direction and rate of interfacial charge transfer. Under the measurement conditions used in this study, Fe–N–C@NiFe-LDH exhibited an ORR half-wave potential (*E*_1/2_) of 0.849 V *vs.* RHE and an OER potential at 10 mA cm^−2^ (*E*_*j*=10_) of 1.61 V, corresponding to an overall potential gap (Δ*E*) of 0.76 V. In a short-term ORR durability test, the composite retained 81.7% of its current after 10 000 s and showed a 23 mV negative shift in *E*_1/2_. In contrast, during OER durability testing, *E*_*j*=10_ shifted positively by 95 mV after 1000 potential cycles, indicating that the initial OER enhancement was not fully maintained. These results highlight the need to balance surface functionalization, preservation of ORR-active structures, NiFe-LDH loading and coverage, and structural durability in the design of Fe–N–C/NiFe-LDH bifunctional oxygen electrocatalysts.

## Abstract 2

Surface modification and NiFe-LDH growth improve OER activity while partially compromising ORR activity, highlighting the importance of balancing surface functionalization, LDH loading, and structural durability.

## Graphical abstract

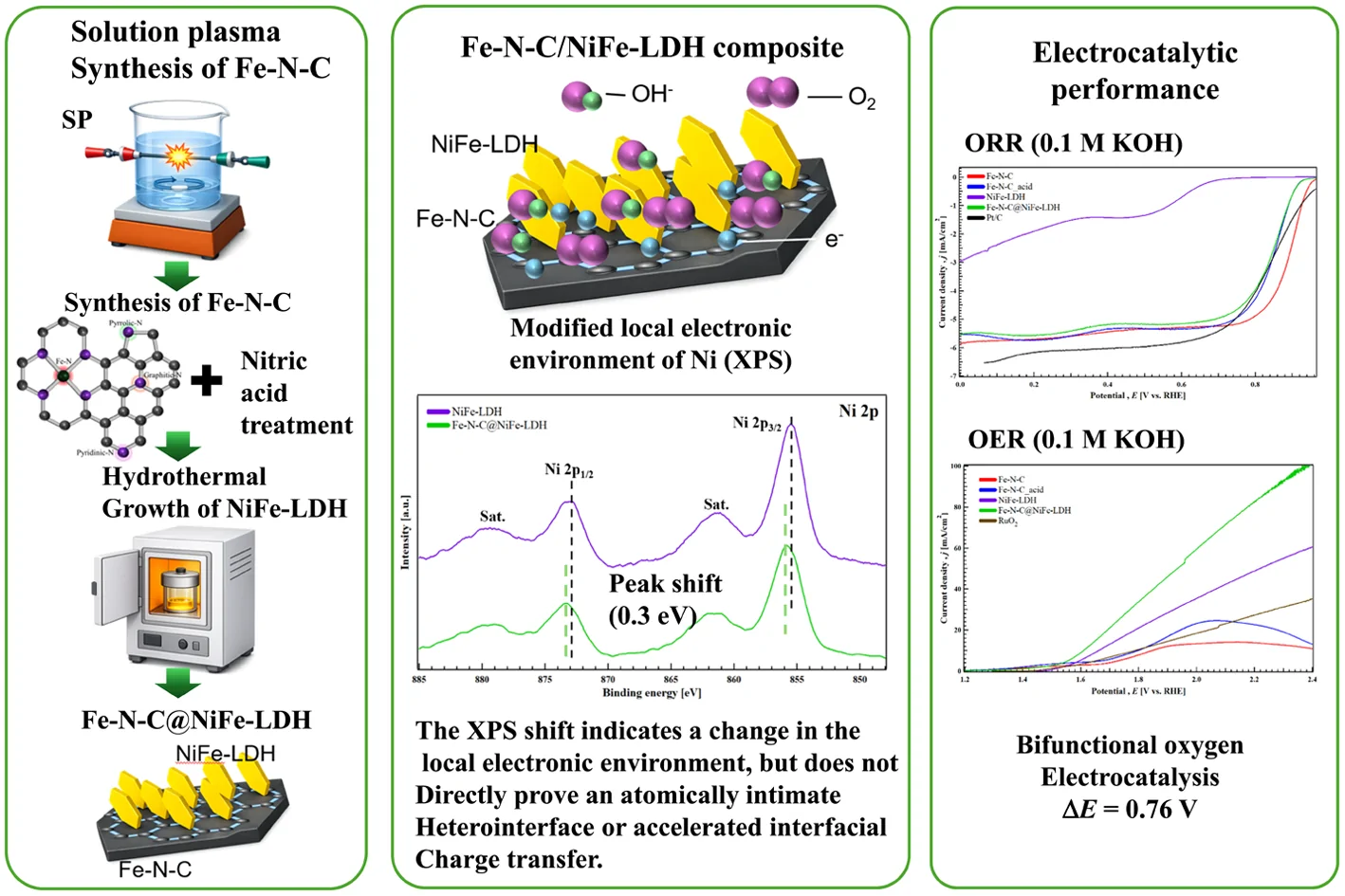

## 1. Introduction

In recent years, the growing demand for a carbon-neutral society and the expanded deployment of renewable energy have accelerated the development of next-generation energy-storage devices with high energy densities. Although lithium-ion batteries are widely used in portable electronics and electric vehicles, their theoretical energy density is inherently limited by the dead weight and space required to store the intercalation-based cathode active materials within the cell. In contrast, metal–air batteries utilize oxygen supplied from the external environment as the cathode active material, eliminating the need to store a large amount of cathode active material within the cell and thereby offering the potential for lightweight systems with unprecedented energy densities. In particular, lithium–air batteries, which employ lightweight lithium metal as the anode and rely on the oxygen reduction and evolution reactions (ORR/OER) at the cathode, have attracted considerable attention as promising next-generation rechargeable batteries.^1,2^ However, several formidable challenges remain before lithium–air batteries can be practically implemented. In particular, the oxygen reduction reaction (ORR) during discharge and the oxygen evolution reaction (OER) during charge at the air electrode are complex processes involving multistep electron transfer and the formation and transformation of reaction intermediates. Consequently, both reactions exhibit sluggish kinetics and require large overpotentials.^3,4^ Slow ORR kinetics decrease the discharge voltage, whereas slow OER kinetics increase the charge voltage, resulting in a large potential gap between discharge and charge. Because this increased voltage hysteresis directly reduces the round-trip energy efficiency of the battery, the development of highly efficient bifunctional oxygen electrocatalysts capable of efficiently promoting both the ORR and OER is essential for improving the performance of lithium–air batteries.^5–7^

Conventionally, platinum (Pt) has been employed as an optimal ORR catalyst, whereas noble-metal oxides such as iridium oxide (IrO_2_) and ruthenium oxide (RuO_2_) exhibit high OER activity. However, these materials are scarce and expensive, imposing significant limitations in terms of resource availability and cost for their large-scale application in energy-storage devices. Accordingly, considerable efforts have recently been devoted to developing noble-metal-free catalysts composed of inexpensive and earth-abundant elements.^8–10^ Among these materials, carbon-based materials are promising platforms for oxygen electrocatalysts owing to their high electrical conductivity, chemical stability, and low density.^11–14^ Nevertheless, pristine carbon materials generally exhibit insufficient ability to adsorb and activate oxygen molecules; therefore, the rational design of active sites through heteroatom doping with nitrogen, phosphorus, or boron, as well as through hybridization with transition metals, is essential.^12,15–17^ In particular, Fe–N–C catalysts composed of iron, nitrogen, and carbon have received considerable attention as among the most promising noble-metal-free ORR electrocatalysts. In Fe–N–C materials, Fe–N_*x*_ moieties embedded within the carbon framework are considered effective active sites for oxygen adsorption, O–O bond activation, and the four-electron reduction pathway.^18–20^ In addition, nitrogen doping alters the electron and spin densities of the carbon framework, thereby synergistically inducing ORR activity in the adjacent carbon atoms.^21,22^ However, most previously reported Fe–N–C catalysts are prepared through high-temperature pyrolysis, an energy-intensive process that frequently suffers from uncontrolled particle aggregation, structural collapse, and non-uniform dopant distributions. In contrast, the solution plasma process, which utilizes non-equilibrium low-temperature plasma generated in a liquid phase, provides a highly localized non-equilibrium reaction field together with rapid quenching. These unique characteristics facilitate the rapid decomposition and reconstruction of precursor molecules, thereby enabling the ultrafast, one-step synthesis of heteroatom-doped carbon materials under non-equilibrium conditions.^23–25^

Although Fe–N–C exhibits excellent ORR activity, its intrinsic OER activity is substantially inadequate to meet the stringent demands of bidirectional energy conversion. Therefore, for application to rechargeable metal–air batteries involving a charging process, an additional materials-design strategy is required to complement its OER activity.^5–7,26–29^ Layered double hydroxides (LDHs) have recently attracted considerable attention as promising materials for enhancing OER activity.^30–32^ LDHs are two-dimensional layered compounds consisting of metal hydroxide layers and interlayer anions, and their catalytic properties can be delicately tuned by controlling the metal composition and interlayer species.^30,31,33^ Among them, NiFe-LDH is a representative noble-metal-free catalyst that exhibits excellent OER activity in alkaline electrolytes.^29^ The synergistic interaction between Ni and Fe is widely recognized to optimize the adsorption energetics of intermediate oxygen species.^34^ Nevertheless, NiFe-LDH intrinsically exhibits poor electrical conductivity, which severely hampers rapid electron transfer during high-rate electrocatalysis.^35,36^ Furthermore, excessive loading of NiFe-LDH can cover the surface of the conductive support, potentially blocking ORR active sites and hindering mass transport.^37,38^ Therefore, rather than simply physically mixing the components, it is desirable to achieve effective nanoscale integration between the conductive carbon support and the OER-active LDH phase. Such integration may facilitate charge transport while limiting excessive coverage of existing ORR-active sites, representing an important consideration in the design of bifunctional oxygen electrocatalysts.^37–40^

In this study, we aimed to construct a bifunctional oxygen electrocatalyst by integrating Fe–N–C synthesized *via* the solution plasma process with NiFe-LDH, thereby combining the ORR activity of the carbon-based component with the OER activity of NiFe-LDH. The Fe–N–C surface was subjected to acid treatment to introduce oxygen-containing functional groups, such as carboxyl and hydroxyl groups, which were expected to act as anchor sites for the heterogeneous nucleation and growth of NiFe-LDH.^41,42^ NiFe-LDH was subsequently grown on the acid-treated Fe–N–C by a hydrothermal process. The effects of acid treatment and subsequent NiFe-LDH growth on the morphology, surface chemical states, ORR/OER activity, and durability were systematically investigated using SEM/EDS, XRD, XPS, and electrochemical measurements. Particular attention was paid to the balance between preserving the ORR-active Fe–N–C structure and introducing the OER-active NiFe-LDH phase. Because the present characterization does not directly resolve nanoscale charge-transfer pathways, interfacial electronic effects are discussed only as possible contributions rather than as an experimentally established mechanism. This study therefore provides insights into the roles of surface functionalization and LDH growth in designing solution-plasma-derived Fe–N–C/NiFe-LDH bifunctional oxygen electrocatalysts.

## 2. Experimental

A schematic illustration depicting the overall synthesis process for the Fe–N–C@NiFe-LDH composite catalyst is presented in Fig. 1.

**Fig. 1:**
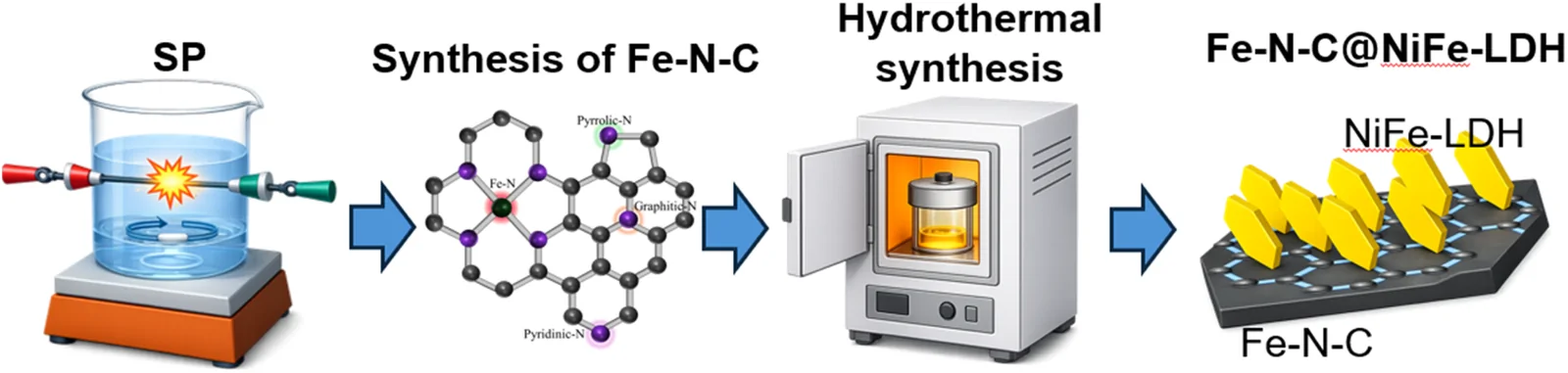
Schematic illustration of the synthesis process for the Fe–N–C@NiFe-LDH composite catalyst, incorporating solution plasma processing (SPP) for Fe–N–C matrix fabrication, acid-mediated surface oxygenation, and subsequent hydrothermal assembly of nanoflower-like NiFe-LDH nanosheets.

### 2.1. Synthesis of Fe–N–C by the solution plasma process

*N*-Methyl-2-pyrrolidone (NMP, Kanto Chemical Co., Inc., >99.7%) was used as the carbon and nitrogen source. Iron phthalocyanine (FePc, Tokyo Chemical Industry Co., Ltd) was used as the iron source. Specifically, FePc (28.4 mg) was dissolved in 100 mL of NMP under vigorous magnetic stirring for 15 min to yield a uniform precursor solution. The resulting precursor solution was then subjected to solution plasma (SP) treatment using the system parameters listed in Table 1. A pair of tungsten (W) electrodes (Nilaco Corp., 1.0 mm in diameter), sheathed in ceramic insulating tubes to confine the discharge region, were arranged in a face-to-face geometry within the SP reaction cell with a fixed electrode gap of 0.50 mm. A bipolar pulsed high-voltage power supply (MPP-HV04, Kurita Seisakusho Co., Ltd) was employed to initiate and maintain the submerged plasma discharge. The discharge was sustained for 6 min at an applied voltage of 3.0 kV, a pulse frequency of 200 kHz, and a pulse width of 1.0 µs. Upon completion of the plasma treatment, the resulting black suspension was collected *via* vacuum filtration using a 0.1 µm polytetrafluoroethylene (PTFE) membrane filter. The retained product was thoroughly washed in sequence with acetone, ethanol, and ultrapure water to remove unreacted organonitrogen precursors and soluble secondary products. The recovered solid was subsequently dried at 100 °C for 12 h in an electric furnace. The dried powder was collected and designated as the Fe–N–C catalyst.

**Table 1:** Solution plasma treatment conditions

Applied voltage	Pulse frequency	Pulse width	Discharge time	Electrode gap
3.0 kV	200 kHz	1.0 µs	6 min	0.50 mm

### 2.2. Acid treatment of Fe–N–C for surface modification

Acid treatment was performed to introduce oxygen-containing functional groups onto the Fe–N–C support surface, thereby providing coordination-active anchoring sites for the localized nucleation and growth of NiFe-LDH. In a typical process, 100 mg of the Fe–N–C powder was dispersed in 100 mL of a 1.0 M HNO_3_ aqueous solution and magnetically stirred at 60 °C for 2 h. After the treatment, the suspension was vacuum-filtered and washed repeatedly with ultrapure water until the filtrate reached neutrality (pH 6.0–7.0). The resulting solid was vacuum-dried at 60 °C for 12 h to yield the surface-functionalized support, designated as Fe–N–C_acid.

### 2.3. Preparation of Fe–N–C@NiFe-LDH composites by hydrothermal synthesis

*In situ* growth of NiFe-LDH on the surface of Fe–N–C_acid was carried out *via* a facile hydrothermal method. Ni(NO_3_)_2_·6H_2_O (FUJIFILM Wako Pure Chemical Corporation) and Fe(NO_3_)_3_·9H_2_O (FUJIFILM Wako Pure Chemical Corporation) were used as the metal precursors, and urea (Hayashi Pure Chemical Ind., Ltd) was employed as a homogeneous precipitant and pH regulator. The amount of urea was maintained at a 20-fold excess relative to the total concentration of metal ions. The precursor compositions for each sample are summarized in Table 2. Predetermined amounts of Fe–N–C_acid and metal salts were dispersed in ultrapure water, then ultrasonically irradiated for 30 min and magnetically stirred for 30 min to ensure a homogeneous suspension. The dispersion was transferred into a Teflon-lined stainless-steel autoclave and heated at 120 °C for 12 h. For comparison, pristine NiFe-LDH was synthesized under identical metal salt concentrations in the absence of Fe–N–C_acid.

**Table 2:** Composition of the precursor solution

Fe–N–C_acid	Ni(NO_3_)_2_·6H_2_O	Fe(NO_3_)_3_·9H_2_O	Urea	Pure water
20 mg	54.5 mg	25.3 mg	300 mg	50 mL

After hydrothermal synthesis, the resulting suspension was cooled naturally to ambient temperature. The resulting product was isolated by centrifugation at 5000 rpm for 10 min. To thoroughly eliminate residual unreacted ions and reaction by-products, the collected precipitate was subjected to multiple washing–centrifugation cycles using ultrapure water and absolute ethanol. The final product was vacuum-dried at 60 °C for 12 h to yield the Fe–N–C@NiFe-LDH composite material.

### 2.4. Material characterization

#### 2.4.1. FE-SEM and EDS

The surface morphology and particle morphology of the samples were observed by field-emission scanning electron microscopy (FE-SEM; JSM-7100F, JEOL Ltd) at an accelerating voltage of 15 kV. The spatial distributions of Fe, Ni, N, C, and O were mapped using energy-dispersive X-ray spectroscopy (EDS; JED-2300F, JEOL Ltd) integrated with the FE-SEM. While EDS provided microscale spatial elemental distribution, the surface electronic states and specific local environments, including the N 1s components assigned to Fe–N_*x*_ active moieties, were rigorously characterized by XPS.

#### 2.4.2. X-ray diffraction (XRD)

The crystalline phases and structural ordering of the samples were determined using an X-ray diffractometer (SmartLab, Rigaku Corporation) equipped with Cu Kα radiation (*λ* = 0.15418 nm), operated at 40 kV and 30 mA. Diffraction patterns were recorded over a 2*θ* range of 5–90° with a step size of 0.01° and a scan rate of 10° min^−1^. Phase identification was carried out by comparing the diffraction peaks with standard crystallographic reference files for FePc, tungsten carbide (WC, evaluated to verify potential electrode-sputtering impurities), and NiFe-LDH.

#### 2.4.3. X-ray photoelectron spectroscopy (XPS)

The surface elemental composition and chemical states were analyzed by X-ray photoelectron spectroscopy (XPS; JPS-9010 MS, JEOL Ltd) utilizing Mg Kα and Al Kα radiation sources. The binding energy scale was calibrated by setting the principal C 1s peak (C–C/C=C bonds) to 284.8 eV. High-resolution core-level spectra of C 1s, N 1s, Ni 2p, and Fe 2p were deconvoluted after applying a Shirley-type background subtraction, using mixed Gaussian–Lorentzian line shapes. Atomic concentrations (at%) were quantified from the survey spectra, whereas the relative ratios of specific chemical bonding states were derived from the integrated peak areas of the deconvoluted high-resolution spectra.

#### 2.4.4. Raman spectroscopy

Raman spectroscopy was performed to evaluate structural defects, degree of graphitization, and phase integrity of the carbon matrix. The characteristic D band (approximately 1350 cm^−1^), G band (approximately 1580 cm^−1^), and 2D band (approximately 2700 cm^−1^) were fitted, and the intensity ratios (*I*_D_/*I*_G_ and *I*_2D_/*I*_G_) were evaluated as structural descriptors. To confirm sample homogeneity and data reproducibility, Raman spectra were acquired from five randomly chosen spots for each sample. The representative averaged values and corresponding spectral fittings are compiled in the SI (Fig. S1 and Table S1).

### 2.5. Electrochemical measurements

#### 2.5.1. Preparation of the working electrode and electrochemical setup

To prepare the catalyst ink, 5 mg of the catalyst powder was dispersed in a mixture of 720 µL of isopropanol, 240 µL of pure water, and 40 µL of a 5 wt% Nafion dispersion (Sigma-Aldrich), followed by tip-ultrasonic dispersion for 1 h to form a well-dispersed catalyst ink. An aliquot (7.5 µL) of the catalyst ink was carefully drop-cast onto a pre-polished glassy carbon rotating disk electrode (RDE; diameter: 4.0 mm; geometric area, *A*_geo_ = 0.126 cm^2^) and dried under ambient conditions to achieve a uniform catalyst loading of approximately 0.30 mg cm^−2^. Electrochemical measurements were conducted at room temperature in a standard three-electrode cell containing an O_2_-saturated 0.1 M KOH aqueous electrolyte (pH ≈ 13.0) using an electrochemical workstation. A Pt wire and a saturated Ag/AgCl electrode were used as the counter and reference electrodes, respectively. All potentials measured against the Ag/AgCl reference were converted to the reversible hydrogen electrode (RHE) scale according to eqn (1):

1 *E*_RHE_ = *E*_Ag/AgCl_ + 0.0591 × pH + *E*^0^_Ag/AgCl_

where *E*^0^_Ag/AgCl_ is the standard potential of the saturated Ag/AgCl reference electrode (0.197 V at 25 °C). The OER overpotential (*η*_OER_) was determined using eqn (2):

2 *η*_OER_ = *E*_RHE_ − 1.23 V

For comparative evaluation under identical conditions, commercial 20 wt% Pt/C and RuO_2_ catalyst electrodes were benchmarked for the ORR and OER, respectively, using the same protocol.

#### 2.5.2. LSV, Tafel analysis, and chronoamperometry (CA)

Linear sweep voltammetry (LSV) and cyclic voltammetry (CV) were performed using an electrochemical analyzer (Model 704 ES, ALS/CH Instruments) equipped with a rotating ring-disk electrode system (RRDE-3A, ALS). For ORR characterization, high-purity O_2_ gas was bubbled into the 0.1 M KOH solution for at least 30 min to ensure complete oxygen saturation. LSV measurements for ORR were performed in the cathodic direction at a rotation speed of 1500 rpm and a scan rate of 10 mV s^−1^. The OER activity was evaluated by sweeping anodically under the same conditions (1500 rpm, 10 mV s^−1^).

For the ORR, the diffusion-limited current density (*j*_L_) was defined as the average current density in the potential range of 0.40–0.55 V *vs.* RHE, and the half-wave potential (*E*_1/2_) was defined as the potential where *j* = *j*_L/2_. To determine the ORR Tafel slope, mass-transport corrections were applied to obtain the kinetic current density (*j*_k_) according to the Koutecký–Levich relationship eqn (3):

3 *j*_k_ = |*j*·*j*_L_/(*j*_L_ − *j*)|

The hydrogen peroxide yield (H_2_O_2_%) and electron transfer number (*n*) during the ORR were determined *via* rotating ring-disk electrode (RRDE) measurements (detailed protocols are provided in Section S1 of the SI).

The OER Tafel slope was extracted from the linear regime of the potential (V *vs.* RHE) *versus* logarithmic current density (log|*j|*) plot in the activation-controlled region. The LSV polarization curves used for the ORR/OER activity comparison were analyzed without post-measurement *iR* correction. The durability of the catalyst toward the ORR was examined *via* chronoamperometric (CA) response in O_2_-saturated 0.1 M KOH at a constant applied potential of −0.264 V *vs.* Ag/AgCl for 10 000 s. To minimize the influence of the initial capacitive and potential-step transient, the reference current density (*j*_0_) used for the current-retention analysis was defined as the average current density measured between 500 and 1000 s. The same normalization procedure was consistently applied to all catalysts. The current retention after 10 000 s was calculated as *j*_10 000_/*j*_0_ × 100, where *j*_10 000_ represents the current density measured at 10 000 s. The durability of the catalysts toward the OER was evaluated by repeated potential cycling using CV over a potential range of 0.235–0.735 V *vs.* Ag/AgCl for up to 1000 cycles. To monitor the evolution of OER activity during cycling, LSV polarization curves were recorded over a potential range of 1.2–1.8 V *vs.* RHE after the 1st, 250th, 500th, 750th, and 1000th CV cycles.

#### 2.5.3. Evaluation of the electric double-layer capacitance (*C*_dl_) and apparent electrochemically active surface area (ECSA)

The apparent electrochemically active surface area (ECSA) was estimated from the electric double-layer capacitance (*C*_dl_) derived in the non-faradaic region. Because a common *C*_s_ value was assumed for materials with different surface compositions, the calculated values are treated as apparent ECSA values and are used primarily for relative comparison among the samples. CV curves were recorded within a potential window of 1.165–1.260 V *vs.* RHE at various scan rates (*ν* = 10, 20, 40, 60, 80, 100 mV s^−1^). At the potential midpoint (1.215 V *vs.* RHE), the capacitive charging current density difference (Δ*j* = *j*_a_ − *j*_c_) was plotted against the scan rate (*ν*), where the slope of Δ*j*/2 *vs. ν* corresponds directly to *C*_dl_. The apparent ECSA was calculated using a standard specific capacitance benchmark (*C*_s_ = 40 µF cm^−2^) and the geometric area (*A*_geo_ = 0.126 cm^2^).^43^ The detailed calculation procedure is provided in Section S2 of the SI. Given that this method assumes a uniform specific capacitance (*C*_s_) across heterogeneous surfaces with different local compositions, the calculated ECSA values are regarded as apparent ECSA values. Accordingly, the ECSA-normalized LSV curves are interpreted as apparent surface-area-normalized activities rather than absolute intrinsic catalytic activities. The corresponding CV profiles, linear *C*_dl_ fittings, and ECSA-normalized LSV polarization curves are compiled in Fig. S3, S4, and Table S3 of the SI.

## 3. Results and discussion

### 3.1. Structural characterization

#### 3.1.1. Morphological observation by FE-SEM

FE-SEM analysis was performed to elucidate the morphological evolution of the catalyst surfaces induced by nitric acid treatment and subsequent hydrothermal hybridization. Representative FE-SEM images of the synthesized materials at various stages are presented in Fig. 2.

**Fig. 2:**
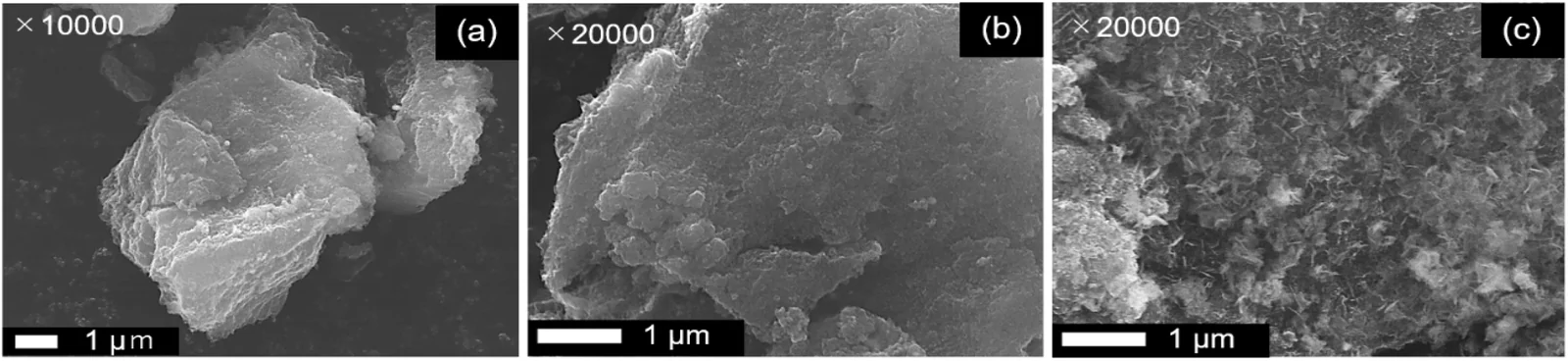
SEM images of (a) pristine Fe–N–C, (b) Fe–N–C_acid after 1.0 M HNO_3_ treatment, and (c) the hybridized Fe–N–C@NiFe-LDH composite, illustrating the surface morphology transformation and the growth of petal-like NiFe-LDH nanosheets on the carbon substrate.

As shown in Fig. 2a, the pristine Fe–N–C synthesized *via* the solution plasma (SP) process exhibited a distinct block-like architecture composed of submicrometer- to micrometer-sized aggregated carbon flakes with smooth outer surfaces. This characteristic morphology is attributed to the ultrafast decomposition, radical recombination, and subsequent thermal quenching of the organic solvent (NMP) and iron precursor (FePc) molecules within the non-equilibrium plasma discharge zone. Following nitric acid modification (Fig. 2b), the Fe–N–C_acid sample displayed pronounced topological roughening, characterized by a higher density of microscale step-edges, nanovoids, and surface defects compared to the untreated counterpart. This structural etching stems from the selective oxidative attack of nitric acid on amorphous carbon domains, reactive graphitic edge planes, and defective sites within the carbon matrix during the thermal acid treatment. Upon subsequent hydrothermal treatment, the resulting Fe–N–C@NiFe-LDH composite (Fig. 2c) exhibited a distinct, highly integrated nanoflower-like architecture that was absent in both Fe–N–C and Fe–N–C_acid. Two-dimensional nanosheets with lateral dimensions in the sub-micrometer regime grew vertically, intersected, and interconnected three-dimensionally to form a porous network over the Fe–N–C_acid substrate. This morphological evolution is consistent with the increased abundance of oxygen-containing functional groups (*e.g.*, carboxyl, carbonyl, and hydroxyl moieties) introduced during the preceding acid treatment. Such oxygenated functionalities can provide coordination and nucleation sites for solvated Ni^2+^ and Fe^3+^ species, thereby potentially facilitating heterogeneous nucleation and subsequent growth of the hydroxide phase on the Fe–N–C_acid surface.^37,38,40^ Collectively, the FE-SEM observations, together with the surface-chemical analysis discussed below, support the interpretation that acid-induced surface functionalization facilitates the growth and dispersion of NiFe-LDH nanosheets on the plasma-derived Fe–N–C support.

#### 3.1.2. Evaluation of elemental distributions by EDS

Energy-dispersive X-ray spectroscopy (EDS) mapping analysis was performed to evaluate the spatial distribution of constituent elements and confirm phase homogeneity across all synthesis stages. Fig. 3–5 display the elemental distributions corresponding to the SEM regions of Fe–N–C, Fe–N–C_acid, and Fe–N–C@NiFe-LDH, respectively.

**Fig. 3:**
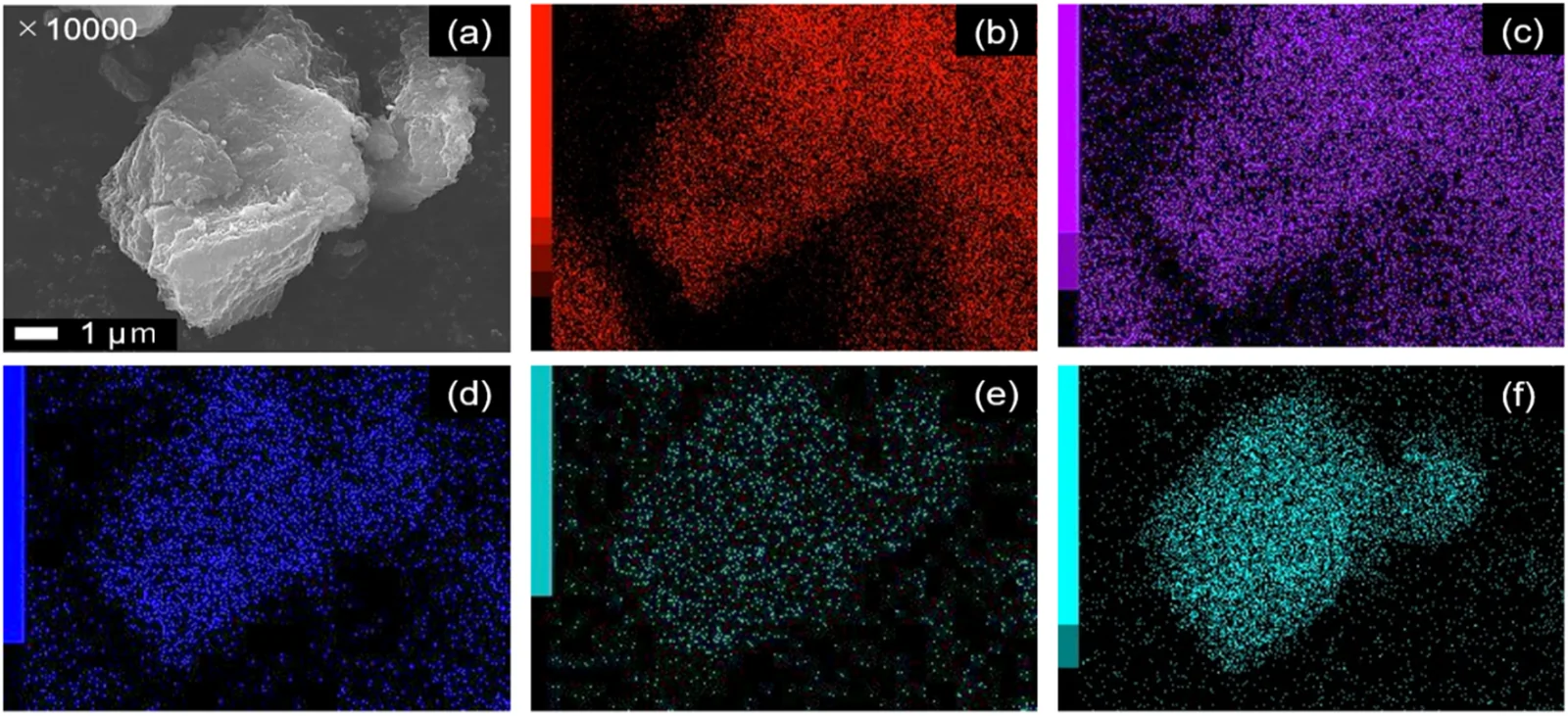
EDS elemental mapping images of the Fe–N–C, demonstrating the spatial distribution of (a) dark-field image, (b) C, (c) N, (d) O, (e) Fe, and (f) W.

**Fig. 4:**
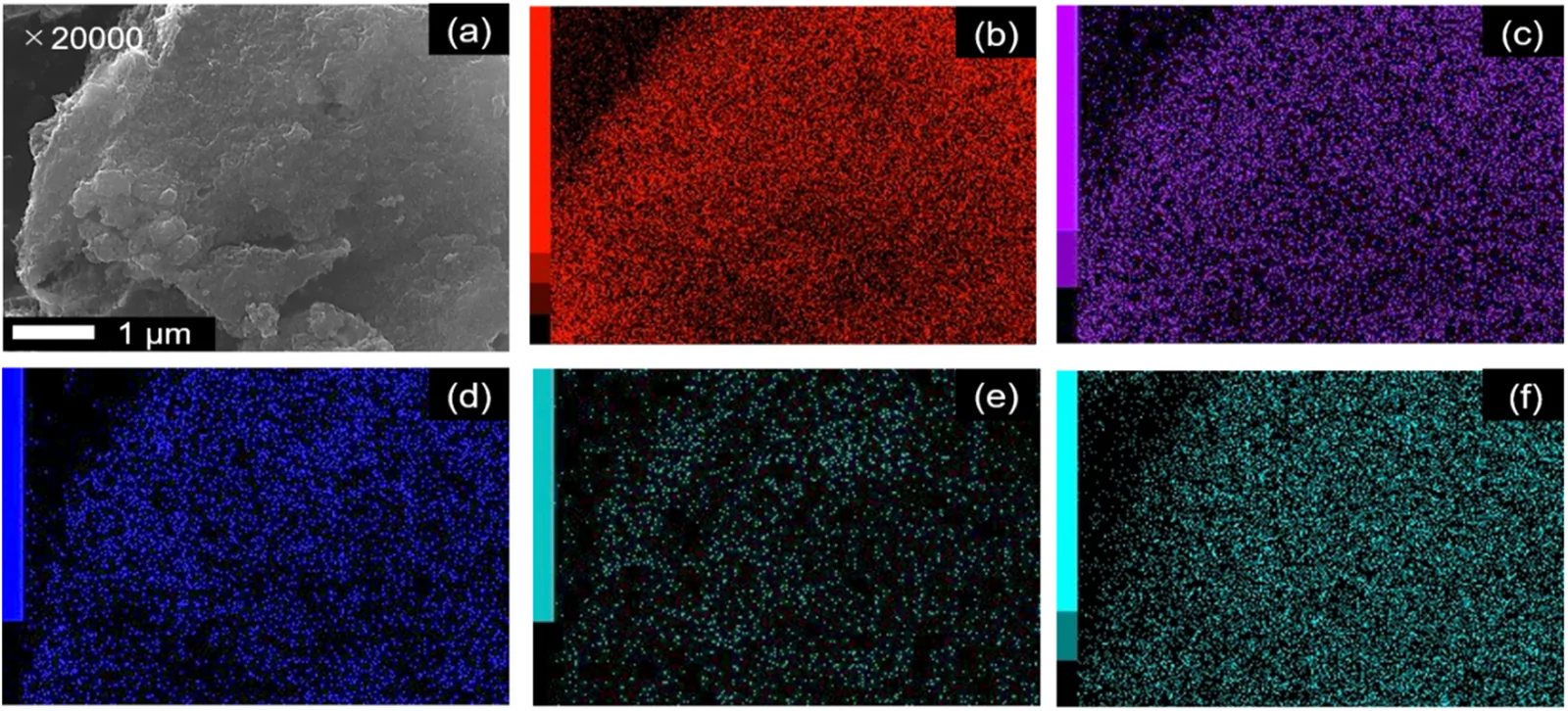
EDS elemental mapping images of Fe–N–C_acid, demonstrating the spatial distribution of (a) dark-field image, (b) C, (c) N, (d) O, (e) Fe, and (f) W.

**Fig. 5:**
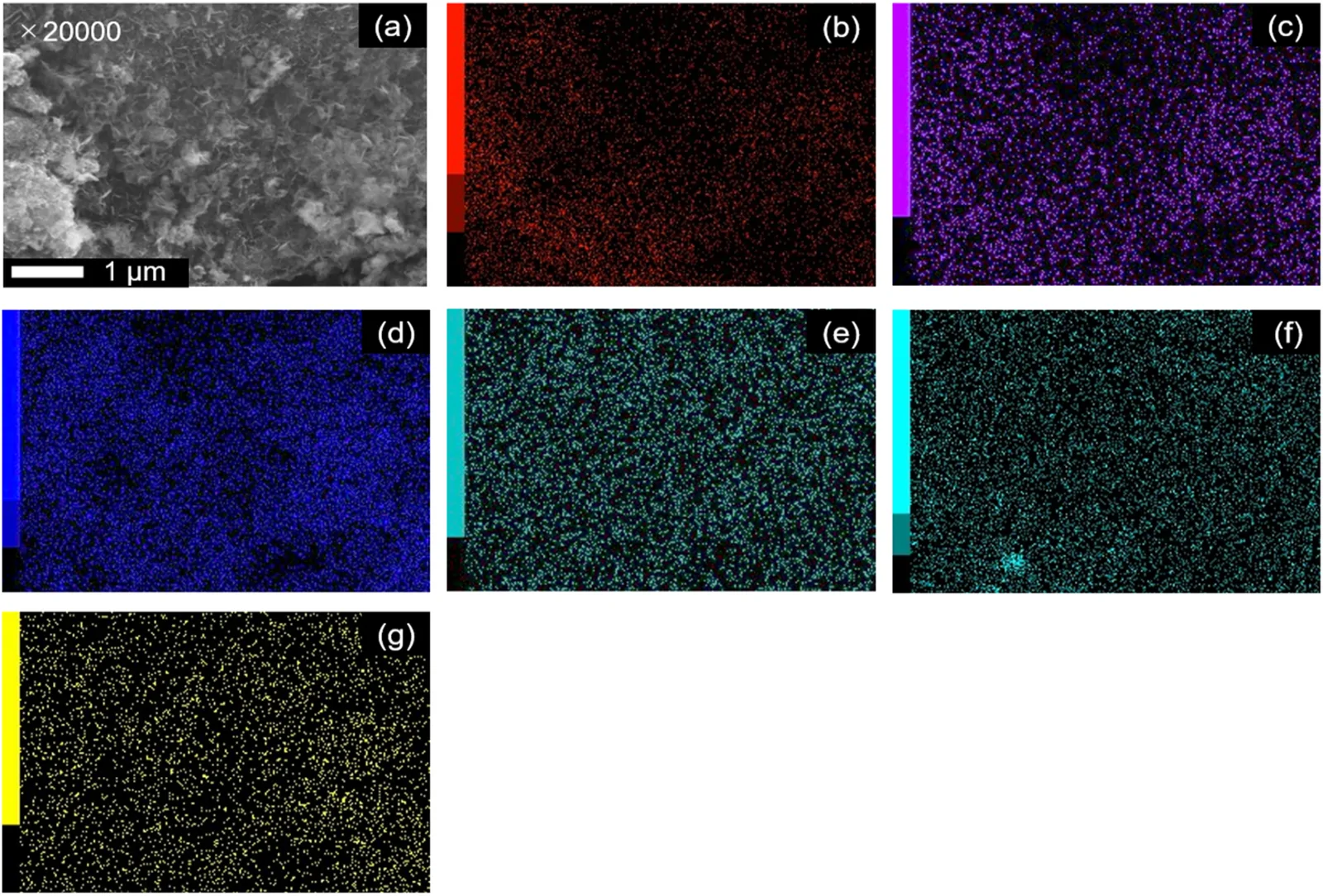
EDS Elemental mapping images of Fe–N–C@NiFe-LDH, demonstrating the spatial distribution of (a) dark-field image, (b) C, (c) N, (d) O, (e) Fe, (f) W, and (g) Ni.

For the untreated Fe–N–C sample (Fig. 3), carbon constitutes the primary matrix, while nitrogen and iron display uniform spatial concurrence across the entire carbon architecture. This high degree of spatial overlap for C, N, and Fe demonstrates that the precursor molecules (FePc and NMP) were rapidly decomposed and homogeneously incorporated into the carbon framework within the highly reactive non-equilibrium plasma zone during the SP process. In contrast, minor localized concentrations of tungsten (W) were detected in isolated regions. This is attributed to physical sputtering and/or micro-erosion of the W electrode tips under high-voltage pulsed discharge, leading to the trace incorporation of W species into the growing carbon matrix. Because the W signal is restricted to localized domains rather than uniformly distributed, it does not represent a dominant phase of the catalyst support. Given that a WC phase originating from the W electrodes was detected by XRD, a possible contribution of electrode-derived W-containing species to the measured electrocatalytic response cannot be excluded. Previous studies have reported that WC alone exhibits limited ORR activity in alkaline media and generally limited OER activity because of its relatively high overpotential.^44,45^ In addition, the W species detected in the present samples were localized by EDS and present only at low surface concentrations according to XPS. Therefore, WC is not expected to be the dominant active phase responsible for the ORR/OER performance observed in this study. Nevertheless, because dedicated WC control experiments were not performed, its possible contribution, including potential promoter or support effects, cannot be completely excluded.

For the acid-treated Fe–N–C_acid sample (Fig. 4), the spatial distributions of C, N, and Fe remained largely unchanged, with no noticeable elemental segregation. This result suggests that the mild nitric acid treatment primarily modified the surface through functionalization and microscale etching without substantially altering the elemental distribution of the Fe–N–C framework.

For the Fe–N–C@NiFe-LDH composite (Fig. 5), Ni, Fe, and O exhibited overlapping spatial distributions that closely corresponded to the nanoflower-like sheet structures observed in the SEM image (Fig. 2c). These observations support the formation and dispersion of a NiFe-LDH phase on the Fe–N–C_acid surface. Concurrently, the relative EDS signals of C and N decreased after hybridization, which can be attributed to the increased contribution of the Ni–Fe–O-rich phase and the partial coverage of the underlying carbon support by NiFe-LDH nanosheets. Taken together, the spatial co-localization of Ni, Fe, and O, together with the observed morphological evolution, supports the formation of a well-dispersed NiFe-LDH phase on the surface-functionalized Fe–N–C matrix.

#### 3.1.3. Crystalline phase analysis by XRD

X-ray diffraction (XRD) measurements were conducted to establish the phase composition, crystallographic integrity, and structural evolution at each synthesis stage. Fig. 6 displays the XRD patterns of pristine Fe–N–C, Fe–N–C_acid, pure NiFe-LDH, and the Fe–N–C@NiFe-LDH composite catalyst.

**Fig. 6:**
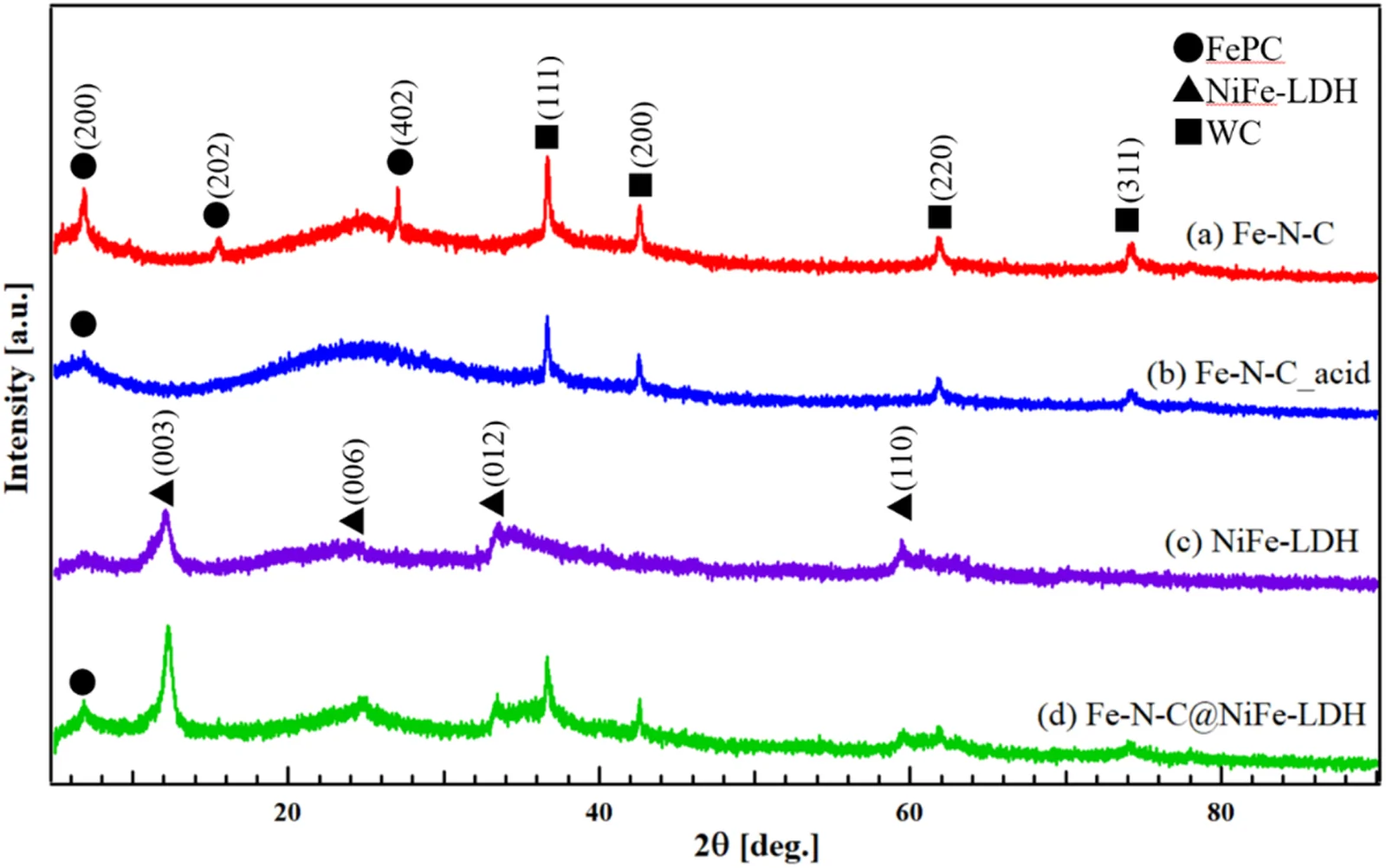
XRD patterns of Fe–N–C, Fe–N–C_acid, pristine NiFe-LDH, and the Fe–N–C@NiFe-LDH composite.

For pristine Fe–N–C (Fig. 6a), a broad diffraction contribution attributable to disordered carbon was observed together with several distinct reflections assigned to residual iron phthalocyanine (FePc, marked with ●) and tungsten carbide (WC, marked with ■). The presence of FePc reflections indicates that a fraction of the FePc precursor remained in a crystalline or partially preserved molecular form after the rapid solution plasma treatment. The reflections assigned to the (111), (200), (220), and (311) planes of WC are attributed to W species introduced by sputtering and/or erosion of the W electrodes during plasma discharge and subsequently reacted with carbon-containing species generated from NMP. This assignment is consistent with the localized W-containing regions detected by EDS. The presence of both residual FePc and WC therefore represents a compositional limitation of the present SPP-derived Fe–N–C material. Because dedicated FePc and WC electrochemical controls were not included, their individual contributions to the measured oxygen electrocatalytic response cannot be quantitatively excluded.

After nitric acid treatment (Fe–N–C_acid, Fig. 6b), the FePc-related diffraction peaks markedly decreased, whereas the WC reflections remained readily detectable. The decrease in the FePc reflections is consistent with partial removal and/or structural disruption of crystalline residual FePc during acid treatment.

Although WC may possess intrinsic electrocatalytic activity, its contribution is expected to be secondary; however, it cannot be quantitatively excluded without dedicated control experiments.

For the Fe–N–C@NiFe-LDH composite (Fig. 6d), newly developed diffraction peaks emerged at 2*θ* ≈ 11.5°, 23.0°, 34.5°, and 59.8°, which are unambiguously indexed to the (003), (006), (012), and (110) planes of hydrotalcite-like NiFe-LDH, respectively (Fig. 6c).^29–31,37^ In particular, the prominent (003) and (006) basal reflections confirm the successful synthesis of a well-ordered, layered double hydroxide phase with regular interlayer spacing on the Fe–N–C_acid surface.

Notably, the NiFe-LDH diffraction features in the composite exhibited broader peaks than those of bulk NiFe-LDH. Such peak broadening may reflect smaller crystallite dimensions, a reduced number of stacked hydroxide layers, or local structural disorder. Together with the nanosheet-like morphology observed by SEM (Fig. 2c), these XRD characteristics suggest that relatively small and highly dispersed NiFe-LDH nanosheets were formed on the Fe–N–C matrix rather than large bulk-like crystallites. Overall, the XRD and SEM results support the successful *in situ* formation of layered NiFe-LDH on the plasma-derived Fe–N–C support.

#### 3.1.4. Surface elemental composition and chemical bonding states by XPS

X-ray photoelectron spectroscopy (XPS) was employed to quantitatively investigate the surface elemental composition and local chemical binding states across the synthesized catalyst interfaces. Fig. 7 illustrates the high-resolution C 1s and N 1s core-level spectra of Fe–N–C and Fe–N–C_acid, alongside the Ni 2p and Fe 2p spectra of pure NiFe-LDH and the Fe–N–C@NiFe-LDH composite. The surface atomic concentrations derived from XPS survey spectra are compiled in Table 3, while the relative deconvoluted peak area fractions for C 1s and N 1s are summarized in Tables 4 and 5, respectively.

**Fig. 7:**
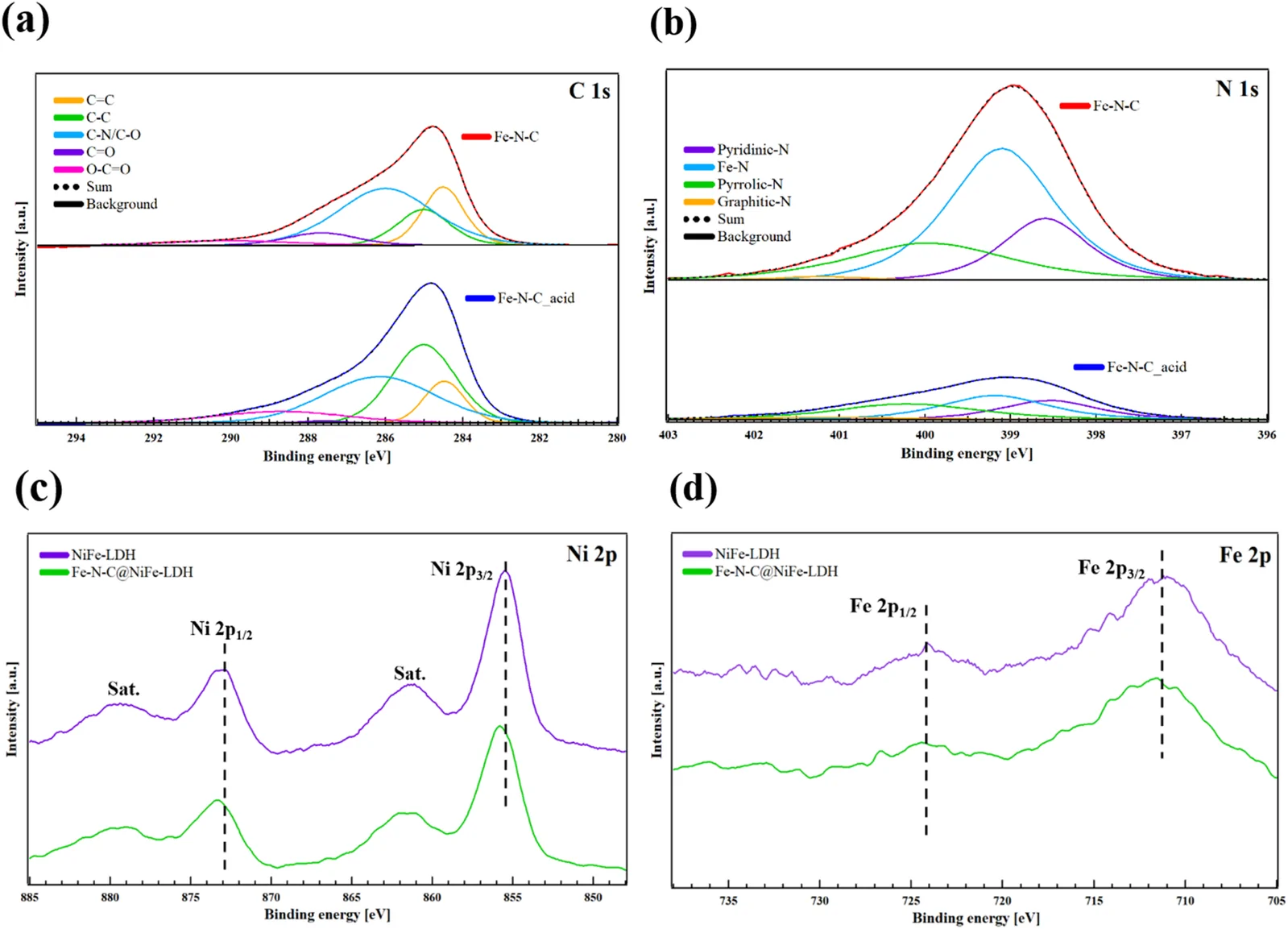
(a) High-resolution XPS C 1s spectra of Fe–N–C and Fe–N–C_acid, (b) high-resolution XPS N 1s spectra of Fe–N–C and Fe–N–C_acid, (c) high-resolution XPS Ni 2p spectra of NiFe-LDH and Fe–N–C@NiFe-LDH, and (d) high-resolution XPS Fe 2p spectra of NiFe-LDH and Fe–N–C@NiFe-LDH.

**Table 3:** Surface atomic concentrations (at%) of the synthesized electrocatalysts determined by XPS survey spectroscopy analysis

Sample	C	N	O	Ni	Fe	W
Fe–N–C	81.1	8.61	9.90	—	0.25	0.15
Fe–N–C_acid	82.7	2.06	14.8	—	0.24	0.23
Fe–N–C@NiFe-LDH	83.4	0.95	14.5	0.82	0.30	0.03

**Table 4:** Relative fractions of C 1s components before and after acid treatment [%]

Sample	C=C	C–C	C–N/C–O	C=O	O–C=O
Fe–N–C	21.6	16.6	48.6	7.88	5.27
Fe–N–C_acid	12.6	37.4	38.7	0.51	10.9

**Table 5:** Relative fractions of N 1s components before and after acid treatment [%]

Sample	Pyridinic-N	Fe–N	Pyrrolic-N	Graphitic-N
Fe–N–C	20.7	55.2	23.1	1.06
Fe–N–C_acid	26.3	36.3	32.2	5.11

The high-resolution C 1s spectra (Fig. 7a) were deconvoluted into five chemical components assigned to sp^2^ graphitic carbon (C=C at 284.5 eV), sp^3^ hybridized carbon (C–C at 285.0 eV), nitrogen/oxygen-bound carbon (C–N/C–O at 286.1 eV), carbonyl groups (C=O at 287.7 eV), and carboxylate species (O–C=O at 288.9 eV). The high-resolution N 1s spectra (Fig. 7b) were fitted with four individual nitrogen configurations: pyridinic-N (398.5 eV), Fe–N coordination (399.4 eV), pyrrolic-N (400.1 eV), and graphitic-N (401.5 eV).^46,47^ These diverse nitrogen moieties confirm the rich doping chemistry typical of plasma-assisted precursor decomposition.^23–25^

As presented in Table 3, pristine Fe–N–C exhibited a surface composition of 81.1 at% C, 8.61 at% N, 9.90 at% O, 0.25 at% Fe, and 0.15 at% W. The N 1s component assigned to Fe–N coordination accounted for 55.2% of the total N 1s envelope (Table 5). After nitric acid treatment, the surface oxygen concentration increased from 9.90 to 14.8 at%, while the relative fraction of the O–C=O component in the C 1s spectrum increased from 5.27% to 10.9% (Table 4). These changes demonstrate that nitric acid treatment increased the abundance of oxygen-containing surface functionalities, particularly carboxyl-related species. Such oxygen-containing groups have been reported to promote the adsorption and coordination of transition-metal cations and can therefore act as nucleation sites during subsequent LDH growth.^41,42^ The SEM and EDS results obtained after hydrothermal synthesis are consistent with this proposed role, although they do not directly identify the individual nucleation sites.

Acid treatment also substantially decreased the surface nitrogen content from 8.61 to 2.06 at% and reduced the relative N 1s contribution assigned to Fe–N coordination from 55.2% to 36.3%. When the total N concentration and the relative Fe–N contribution are considered together, the apparent surface contribution assigned to Fe–N species decreased from approximately 4.75 to 0.75 at%. This decrease should not be interpreted as a direct quantitative measurement of all catalytically active Fe–N_*x*_ sites; nevertheless, it clearly demonstrates a substantial loss of Fe–N-related surface species after acid treatment. The XPS results therefore reveal a trade-off in which nitric acid treatment increases oxygen-containing functionalities favorable for subsequent LDH nucleation while simultaneously decreasing N-containing and Fe–N-related surface species associated with ORR activity.

In the high-resolution Ni 2p (Fig. 7c) and Fe 2p (Fig. 7d) spectra, pristine NiFe-LDH and Fe–N–C@NiFe-LDH exhibited the characteristic core-level features of Ni- and Fe-containing hydroxide phases, together with their corresponding satellite structures.^29,34^ Relative to pristine NiFe-LDH, the Ni 2p core level of Fe–N–C@NiFe-LDH exhibited a positive binding-energy shift of approximately +0.3 eV. This shift indicates that the local chemical/electronic environment of Ni was modified after composite formation. Such a change is consistent with electronic perturbation associated with contact between the NiFe-LDH phase and the Fe–N–C-based support. However, the binding-energy shift alone does not demonstrate an atomically intimate or structurally well-defined heterointerface, nor does it establish the direction or rate of charge transfer between the two components. Accordingly, the XPS result is interpreted as evidence of a modified local electronic environment upon composite formation rather than definitive proof of accelerated interfacial electron transfer.

In summary, XPS analysis showed that nitric acid treatment increased oxygen-containing surface functionalities while decreasing the surface N content and Fe–N-related N 1s contribution. These surface changes are consistent with the subsequent growth of NiFe-LDH observed by SEM/EDS and XRD. In addition, the Ni 2p binding-energy shift after composite formation indicates a change in the local electronic environment of the NiFe-LDH phase. Therefore, optimization of the acid-treatment conditions remains important for promoting LDH nucleation while minimizing the loss of ORR-relevant Fe–N-related surface species.

### 3.2. Evaluation of electrocatalytic performance by electrochemical measurements

#### 3.2.1. Evaluation of ORR and OER performance by LSV

Linear sweep voltammetry (LSV) measurements using a rotating disk electrode (RDE) setup were performed to systematically evaluate the electrocatalytic performance of the synthesized materials toward both the oxygen reduction reaction (ORR) and oxygen evolution reaction (OER). Electrochemical durability was further assessed by chronoamperometry (CA) and accelerated durability tests (ADTs) involving repeated potential cycling using cyclic voltammetry (CV). Fig. 8 and 9 display the ORR and OER polarization curves, Tafel plots, CA results, and polarization curves recorded before and after the potential-cycling durability tests for the respective samples. Table 6 summarizes key performance metrics, including the ORR half-wave potential (*E*_1/2_), the potential required to achieve an OER current density of 10 mA cm^−2^ (*E*_*j*=10_), and the potential gap between these two values (Δ*E*). As an integrated figure-of-merit for overall bifunctional oxygen electrocatalysts, Δ*E* was calculated according to eqn (4).

4 Δ*E* = *E*_*j*=10_ − *E*_1/2_

where Δ*E* is an integrated performance metric that reflects the overpotentials associated with both the ORR and OER; a smaller Δ*E* indicates superior overall energy efficiency with reduced overpotentials for both reactions on the same catalyst.^5–7,37^

**Fig. 8:**
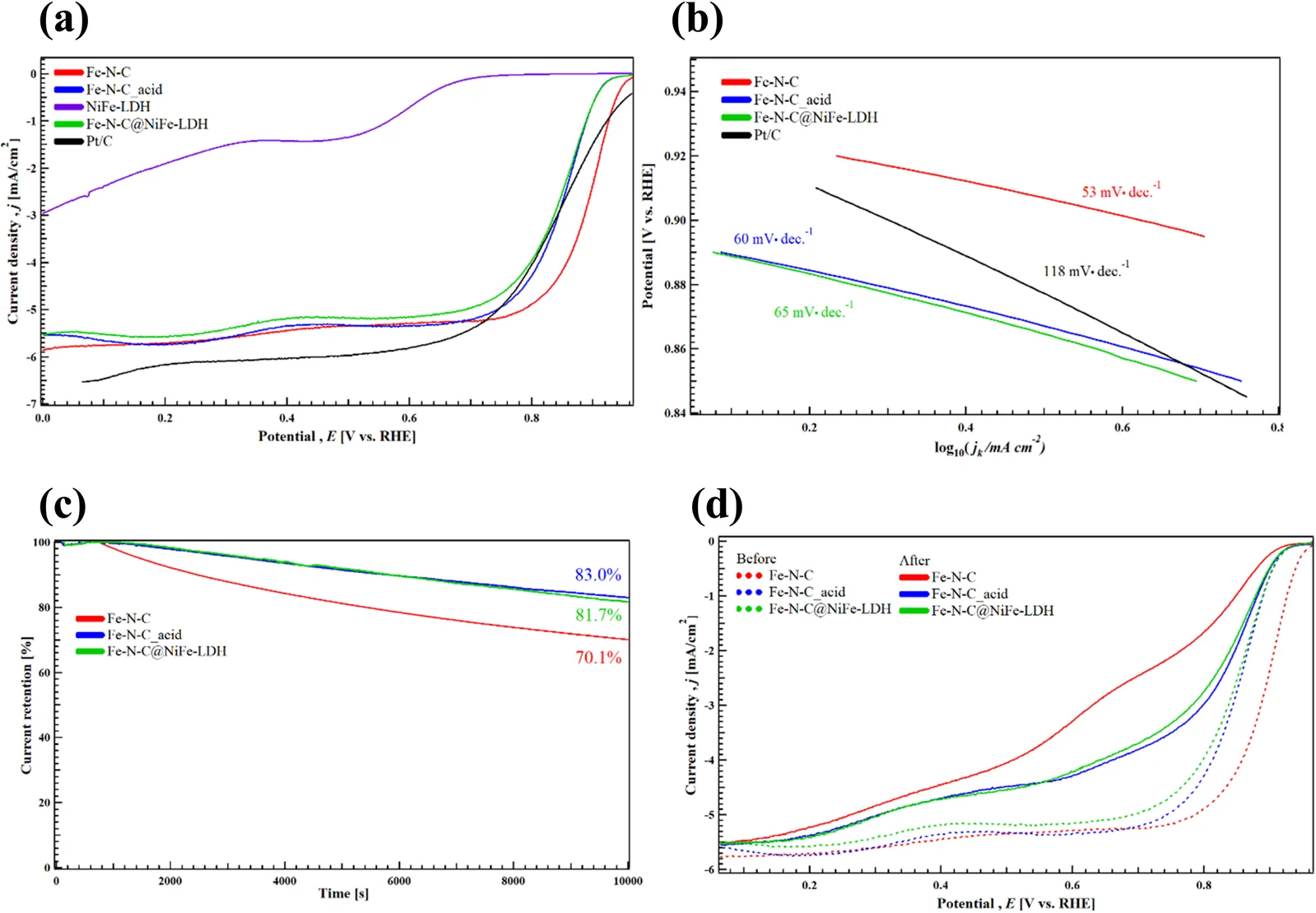
ORR electrocatalytic performance in O_2_-saturated 0.1 M KOH at 1500 rpm: (a) LSV polarization curves, (b) corresponding Tafel plots, (c) chronoamperometric (*i*–*t*) responses recorded for 10 000 s at 0.70 V *vs.* RHE and (d) ORR polarization curves before and after the CA durability test for Fe–N–C, Fe–N–C_acid, Fe–N–C@NiFe-LDH, and commercial 20 wt% Pt/C.

**Fig. 9:**
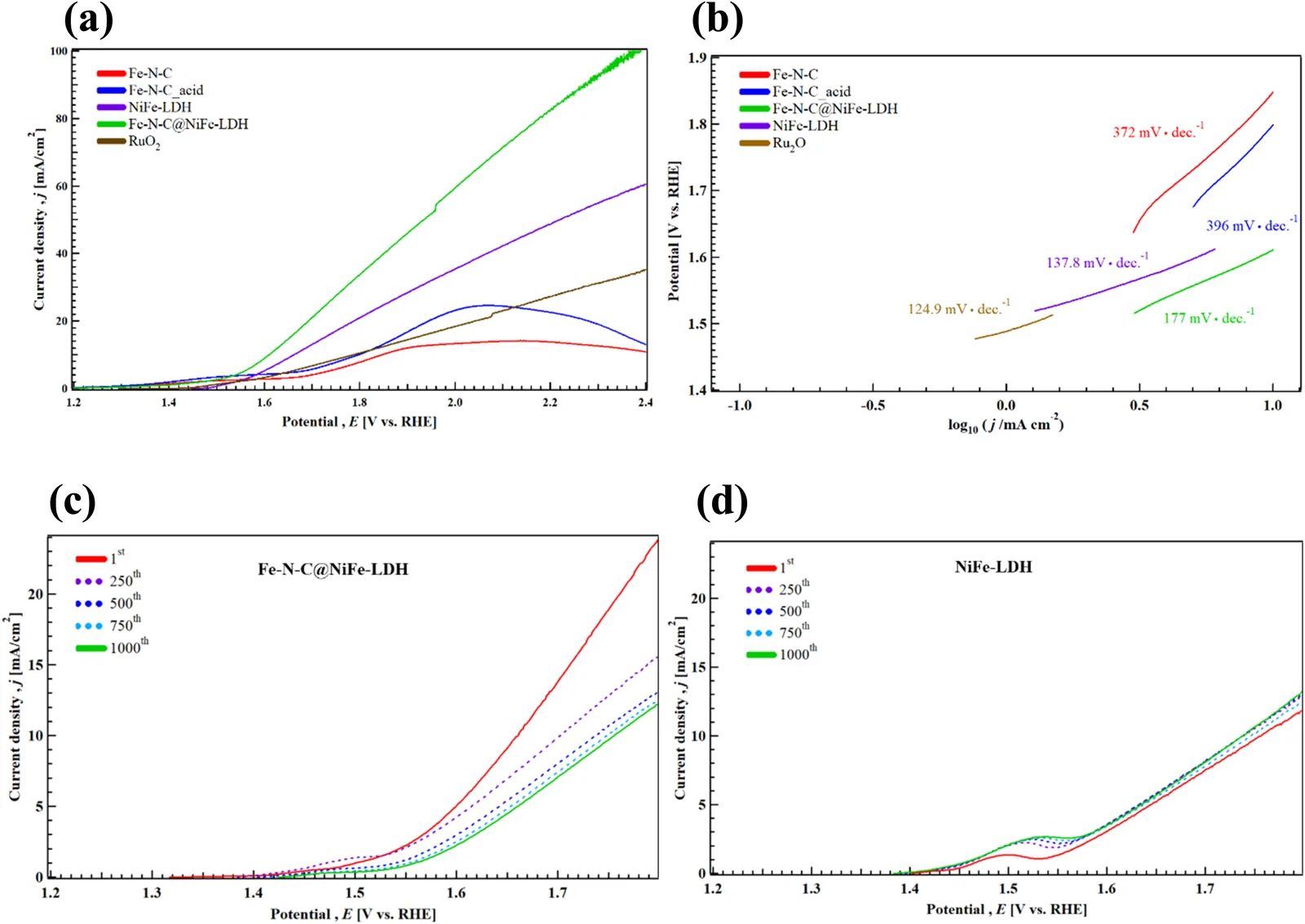
OER electrocatalytic performance in 0.1 M KOH at 1500 rpm: (a) LSV polarization curves and (b) corresponding Tafel plots for Fe–N–C, Fe–N–C_acid, pristine NiFe-LDH, Fe–N–C@NiFe-LDH, and benchmark RuO_2_. (c) OER polarization curves of Fe–N–C@NiFe-LDH during the potential-cycling durability test and (d) OER polarization curves of NiFe-LDH during the potential-cycling durability test.

**Table 6:** Summary of electrocatalytic performance parameters for ORR and OER (*E*_1/2_, *E*_*j*=10_, and bifunctional potential gaps Δ*E*) of the synthesized catalysts and commercial reference materials (20 wt% Pt/C and RuO_2_) in 0.1 M KOH

Sample	*E*_1/2_ [V *vs.* RHE]	*E*_*j*=10_ [V *vs.* RHE]	Δ*E* [V]
Fe–N–C	0.894	1.85	0.96
Fe–N–C_acid	0.852	1.80	0.95
NiFe-LDH	0.315	1.66	1.35
Fe–N–C@NiFe-LDH	0.849	1.61	0.76
Pt/C	0.843	—	—
RuO_2_	—	1.79	—

Untreated Fe–N–C exhibited a half-wave potential (*E*_1/2_) of 0.894 V *vs.* RHE, demonstrating exceptional intrinsic ORR activity with a more positive *E*_1/2_ than that of the benchmark commercial 20 wt% Pt/C catalyst (0.843 V *vs.* RHE). This high ORR activity is attributed to Fe–N_*x*_ coordination structures efficiently incorporated into the carbon framework during the solution plasma process, which effectively promote O_2_ chemisorption and facilitate the cleavage of O–O bonds. In addition, nitrogen species such as pyridinic-N and graphitic-N modulate the local electronic structure of adjacent carbon atoms, further enhancing overall ORR performance.^18,19^

In contrast, *E*_1/2_ of Fe–N–C_acid shifted negatively to 0.852 V *vs.* RHE after acid treatment. As discussed in the XPS analysis (Section 3.1.4), acid treatment increased the density of surface oxygen-containing functional groups (O–C=O), while simultaneously inducing competitive leaching and degradation of surface nitrogen and active Fe–N_*x*_ coordination species. Therefore, although acid treatment introduced oxygen-containing functional groups that can serve as essential nucleation sites for NiFe-LDH, the decrease in surface Fe–N_*x*_-related species is consistent with, and likely contributes to, the observed attenuation of ORR activity. In addition, Fe–N–C_acid alone did not exhibit sufficient OER activity.

Conversely, pristine NiFe-LDH exhibited a low *E*_1/2_ of 0.315 V *vs.* RHE, reflecting severely sluggish ORR activity due to the intrinsic lack of ORR-active centers and poor electrical conductivity. In contrast, NiFe-LDH exhibited an *E*_*j*=10_ of 1.66 V *vs.* RHE (corresponding to an overpotential of *η* = 0.43 V), which was lower than that of the noble-metal-based RuO_2_ catalyst (1.79 V *vs.* RHE; *η* = 0.56 V). This high OER activity is attributed to the *in situ* generation of active Ni/Fe oxyhydroxide species under anodic potentials, which accelerate the intermediate adsorption and conversion steps (OH*, O*, and OOH*).^29,34^ Nevertheless, because of its extremely poor ORR activity, pristine NiFe-LDH exhibited a large bifunctional gap (Δ*E*) of 1.35 V.

In contrast, the Fe–N–C@NiFe-LDH composite successfully coupled both catalytic functions, achieving an *E*_1/2_ of 0.849 V *vs.* RHE for the ORR. Although this value was 45 mV lower than that of pristine Fe–N–C, the composite maintained ORR activity comparable to or slightly exceeding that of Pt/C (*E*_1/2_ = 0.843 V *vs.* RHE). Furthermore, the *E*_*j*=10_ for the OER decreased to 1.61 V *vs.* RHE (overpotential of 380 mV), which was 50 mV lower than pristine NiFe-LDH and 180 mV lower than RuO_2_. Consequently, Fe–N–C@NiFe-LDH achieved a Δ*E* of 0.76 V, substantially smaller than those of Fe–N–C (0.96 V), Fe–N–C_acid (0.95 V), and pristine NiFe-LDH (1.35 V).

The *E*_1/2_ decreased by 42 mV, from 0.894 V for Fe–N–C to 0.852 V after acid treatment. In contrast, the Fe–N–C@NiFe-LDH composite exhibited an *E*_1/2_ of 0.849 V, corresponding to only a 3 mV decrease relative to Fe–N–C_acid. Therefore, most of the observed loss in ORR activity is considered to originate from the acid-treatment step rather than from the subsequent NiFe-LDH growth process.

In contrast, the Fe–N–C@NiFe-LDH composite exhibited improved OER activity relative to pristine NiFe-LDH. This improvement may be associated with the conductive Fe–N–C substrate, which can provide an effective electron-transport framework, together with changes in the local electronic environment induced by composite formation. The +0.3 eV binding-energy shift observed in the Ni 2p spectrum supports a modification of the local electronic state of Ni sites upon contact with Fe–N–C. However, this XPS shift alone does not establish the direction or rate of interfacial charge transfer, and therefore the contribution of interfacial electronic effects to the enhanced OER activity cannot be quantitatively determined from the present data.^29,34,37,40^

Therefore, further optimization of bifunctional ORR/OER performance should focus on controlling the thickness and dispersion of NiFe-LDH nanosheets while maintaining sufficient exposure of the underlying ORR-active sites and favorable interfacial contact.

The ORR Tafel slopes (Fig. 8b) of Fe–N–C, Fe–N–C_acid, and Fe–N–C@NiFe-LDH were 53, 60, and 65 mV dec^−1^, respectively, demonstrating that the favorable electron-transfer kinetics characteristic of the Fe–N–C matrix were largely preserved upon hybridization. For Pt/C, the Tafel slope in the selected high-potential region was 118 mV dec^−1^. Although the absolute Tafel slope depends on multiple operational factors, including fitting range, mass-transport correction, and catalyst layer conditions, and therefore should not be used in isolation to deduce precise mechanism, these findings confirm that the ORR kinetics were not severely disrupted by composite formation.

In contrast, the OER Tafel slopes (Fig. 9b) were 372, 396, 138, 177, and 125 mV dec^−1^ for Fe–N–C, Fe–N–C_acid, pristine NiFe-LDH, Fe–N–C@NiFe-LDH, and RuO_2_, respectively. Although the Tafel slope of the composite was substantially lower than those of the carbon-based samples, it remained higher than those of pure NiFe-LDH and RuO_2_. This behavior indicates that the composite exhibits favorable OER polarization at relatively low current densities, whereas increasingly pronounced kinetic and resistive losses emerge as the current density increases. These limitations are likely associated with increased ionic/electronic resistance within the LDH-containing catalyst layer, restricted mass transport through the restricted reactant/product transport within the composite catalyst layer, and hampered detachment of generated oxygen gas bubbles.

The short-term ORR durability of Fe–N–C, Fe–N–C_acid, and Fe–N–C@NiFe-LDH was evaluated by chronoamperometry for 10 000 s in O_2_-saturated 0.1 M KOH (Fig. 8c). To minimize the influence of the initial capacitive and potential-step transient, the average current recorded between 500 and 1000 s was used as the reference current for normalization, and the same procedure was applied to all three catalysts. Based on this definition, the current retentions after 10 000 s were 70.1%, 83.0%, and 81.7% for Fe–N–C, Fe–N–C_acid, and Fe–N–C@NiFe-LDH, respectively. The higher current retention of Fe–N–C_acid and Fe–N–C@NiFe-LDH compared with untreated Fe–N–C suggests that the acid-treatment step improves the short-term electrochemical stability of the Fe–N–C-derived materials. However, because the composite was prepared from the acid-treated Fe–N–C precursor, the individual contribution of the subsequently deposited NiFe-LDH phase to the improved current retention cannot be isolated from the present data.

Consistent with the chronoamperometric results, the ORR polarization curves recorded before and after the durability test showed markedly different degrees of activity loss among the catalysts (Fig. 8d). The *E*_1/2_ of untreated Fe–N–C decreased from 0.894 to 0.757 V, corresponding to a negative shift of 137 mV, whereas Fe–N–C_acid exhibited a substantially smaller shift from 0.852 to 0.837 V (−15 mV). Fe–N–C@NiFe-LDH showed an intermediate shift from 0.849 to 0.826 V (−23 mV). These results indicate that both Fe–N–C_acid and Fe–N–C@NiFe-LDH exhibit improved short-term ORR stability relative to untreated Fe–N–C. Nevertheless, the 18.3% current loss and 23 mV negative shift in *E*_1/2_ observed for Fe–N–C@NiFe-LDH demonstrate that measurable ORR degradation still occurs during the 10 000 s test.

The OER durability of Fe–N–C@NiFe-LDH and pristine NiFe-LDH was evaluated by repeated potential cycling for 1000 cycles. As shown in Fig. 9c and d, Fe–N–C@NiFe-LDH exhibited a progressive decrease in OER activity with increasing cycle number. The *E*_*j*=10_ increased from 1.661 V at the 1st cycle to 1.756 V after 1000 cycles, corresponding to a positive shift of 95 mV. In contrast, pristine NiFe-LDH exhibited a slight improvement in OER activity during repeated cycling, with *E*_*j*=10_ decreasing from 1.758 to 1.739 V after 1000 cycles, corresponding to an improvement of 19 mV.

The modest activation observed for pristine NiFe-LDH is consistent with the dynamic electrochemical reconstruction widely reported for NiFe-based hydroxides under anodic OER conditions. NiFe-LDH can behave as a precatalyst that undergoes potential-induced deprotonation and oxidation toward catalytically active Ni(Fe) oxyhydroxide species. Recent time-resolved *in situ* X-ray absorption spectroscopy has demonstrated that Fe incorporation facilitates deprotonation of bridged Ni–OH–Fe groups in NiFe-LDH, promoting the transformation toward Ni(Fe)OOH and the formation of high-valence Ni^3+*δ*^ species under OER conditions.^54^*Operando* Raman spectroscopy and *ex situ* characterization have also shown that surface reconstruction of NiFe-LDH-derived materials into oxyhydroxide species can enhance OER activity by modifying the adsorption energetics of reaction intermediates.^55^ More recently, mixed-valence NiFe-LDH has been reported to reconstruct into α/δ-FeOOH/γ-NiOOH phases during OER activation, further illustrating the dynamic nature of NiFe-LDH under anodic polarization.^56^ Accordingly, the 19 mV improvement observed for pristine NiFe-LDH in the present study may reflect progressive electrochemical activation associated with such surface reconstruction. However, because post-cycling structural or spectroscopic characterization was not performed in the present study, formation of a specific oxyhydroxide phase cannot be directly established, and this interpretation should therefore be regarded as a plausible explanation rather than definitive mechanistic evidence.

The contrasting durability trends of pristine NiFe-LDH and Fe–N–C@NiFe-LDH are particularly noteworthy. Whereas pristine NiFe-LDH showed modest electrochemical activation, the composite exhibited a progressive 95 mV increase in *E*_*j*=10_. This difference suggests that the activity loss of the composite cannot be explained simply by reconstruction of the NiFe-LDH phase itself. Rather, degradation processes associated with the composite architecture may outweigh any beneficial electrochemical activation of the LDH component. Possible contributions include oxidative degradation of the carbon support at anodic OER potentials, deterioration or partial loss of electrical contact between NiFe-LDH and Fe–N–C, and structural or morphological changes within the composite catalyst layer that increase electrical and/or mass-transport resistance. Thus, although electrochemical reconstruction of NiFe-LDH may generate more OER-active oxyhydroxide-like surface species, preservation of the conductive Fe–N–C/LDH architecture appears to be an important factor governing the durability of the present composite. Further stabilization of the catalyst/support contact is therefore required to preserve the initial OER enhancement during repeated potential cycling.

#### 3.2.2. Correlation between structure and electrocatalytic performance

The results obtained from structural and compositional analyses (SEM, EDS, XRD, XPS, and Raman spectroscopy) and electrochemical measurements (LSV, RRDE, and CA) were comprehensively evaluated to elucidate the relationship between the surface chemical states and interfacial structures, and the resulting oxygen reduction reaction (ORR) and oxygen evolution reaction (OER) catalytic activities.

Raman spectroscopy was employed to evaluate changes in the carbon framework structure following chemical modification and hybrid synthesis (Fig. S1 and Table S1). The intensity ratio of the D band to G band (*I*_D_/*I*_G_) was 1.13 for untreated Fe–N–C and 1.12 for Fe–N–C_acid after acid treatment, showing negligible variation. This result suggests that nitric acid treatment under the conditions employed in this study did not substantially disrupt the bulk carbon framework, thereby preserving the overall defect density and domain size of the graphitic backbone.^48^ In contrast, Fe–N–C@NiFe-LDH exhibited a lower *I*_D_/*I*_G_ value of 0.99 and a higher 2D-to-G band intensity ratio (*I*_2D_/*I*_G_) of 0.32 compared with Fe–N–C and Fe–N–C_acid. The simultaneous decrease in (*I*_D_/*I*_G_) and increase in (*I*_2D_/*I*_G_) suggest an increased relative contribution from more ordered or graphitic carbon domains after composite formation. However, because NiFe-LDH nanosheets cover the Fe–N–C surface, the Raman scattering environment and the relative contribution of different carbon domains may also be altered. Therefore, these changes should not be interpreted as direct evidence of graphitization induced by the hydrothermal treatment, but rather as an apparent increase in the contribution of structurally ordered carbon domains in the composite.

Furthermore, the H_2_O_2_ yield and electron transfer number (*n*) determined from rotating ring-disk electrode (RRDE) measurements are shown in Fig. S2 and Table S2. Fe–N–C exhibited an H_2_O_2_ yield of less than 0.01% and an electron transfer number of 4.00. Even after acid treatment and hybridization with NiFe-LDH, the H_2_O_2_ yields of Fe–N–C_acid and Fe–N–C@NiFe-LDH remained low at 2.46% and 2.54%, respectively, while the electron transfer numbers were calculated to be 3.95 for both samples. These quantitative results indicate that, despite the decrease in surface Fe–N_*x*_ species caused by acid treatment (XPS) and partial physical coverage by LDH nanosheets (SEM), the ORR on the composite catalyst predominantly proceeds through a highly efficient four-electron (4e^−^) reduction pathway with minimal peroxide generation.

The apparent ECSA, estimated from the electric double-layer capacitance (*C*_dl_), decreased sequentially from Fe–N–C (4.63 mF cm^−2^/14.5 cm^2^) to Fe–N–C_acid (3.56 mF cm^−2^/11.2 cm^2^) and Fe–N–C@NiFe-LDH (2.46 mF cm^−2^/7.72 cm^2^) (Fig. S3 and Table S3). Thus, no simple positive correlation was observed between the apparent ECSA and ORR activity. This phenomenon can be explained by two distinct mechanisms:

1. For Fe–N–C_acid: acid treatment selectively removed low-activity amorphous carbon species and residual metallic impurities from the outermost surface. Although this cleaning process reduced the net macroscopic surface area (*C*_dl_), it effectively unmasked high-density Fe–N_*x*_ centers, improving molecular-level reactant accessibility (O_2_ diffusion).

2. For Fe–N–C@NiFe-LDH: although the hydroxide surface introduced by NiFe-LDH contributes to double-layer charging, the physical shielding of underlying Fe–N_*x*_ active sites by overgrown, dense LDH nanosheets reduced the accessible ORR site density, accounting for the slight decrease in ORR performance.

Therefore, although changes in the apparent current density per unit surface area were observed in the ECSA-normalized specific-activity evaluation (Fig. S4), the actual catalytic performance of this system, particularly its bifunctional ORR/OER activity, cannot be explained solely by the apparent surface area or the magnitude of *C*_dl_. A comprehensive evaluation incorporating chemical oxidation states, active-site accessibility (Fe–N_*x*_*vs.* Ni/Fe species), interfacial electronic coupling, and local mass-transport kinetics is essential. It should also be noted that the comparison shown in Fig. S4 represents an apparent activity metric calculated by assuming a common specific capacitance (*C*_s_) for all samples. Because *C*_s_ differs substantially between conductive carbon frameworks and dielectric transition-metal hydroxides, these ECSA-normalized values serve as qualitative indicators rather than absolute measures of intrinsic active-site turnover.

Based on the above findings, achieving superior bifunctional activity (low Δ*E* and high 4e^−^ selectivity) in carbon/metal-hydroxide hybrid catalysts requires the rational design of coupled interfaces where multiple functional factors act synergistically:

• Acid pretreatment: acid washing cleanses the carbon surface by removing low-activity amorphous phases, thereby enhancing the intrinsic accessibility of embedded active centers. However, acid concentration and exposure time must be precisely controlled to minimize the competitive degradation of primary Fe–N_*x*_ active centers.

• Morphology & coverage control: to maximize OER capability without sacrificing ORR performance, NiFe-LDH must be grown as ultrathin, highly dispersed nanosheets. This prevents severe physical blockage of the underlying Fe–N–C framework, facilitating unhindered mass transport of O_2_ and OH^−^ reactants.

• Interfacial electronic environment: the binding-energy shift observed by XPS suggests that composite formation modifies the local electronic environment of the NiFe-LDH phase through interactions with the Fe–N–C substrate. Such interfacial electronic effects may influence the electrocatalytic properties of the composite; however, the present data do not directly establish accelerated electron transfer or changes in the adsorption energies of OER intermediates.

• Interfacial mechanical robustness: suppressing interfacial delamination and phase transformation during long-term polarization is crucial for ensuring operational durability.

Although the half-cell measurements demonstrate bifunctional ORR/OER activity and the XPS results suggest changes in the local electronic environment upon composite formation, evaluating full-cell metal–air battery performance under practical operating conditions (*e.g.*, charge–discharge cycling, power density, and rate capability) was beyond the scope of the present study. Further system-level optimization, including gas-diffusion layer engineering, binder selection, and electrolyte membrane compatibility, will be systematically addressed in future work.

#### 3.2.3. Comparison of bifunctional performance with recently reported catalysts

To objectively evaluate the bifunctional oxygen electrocatalytic performance of the Fe–N–C@NiFe-LDH composite catalyst developed in this study, its representative activity metrics (*E*_1/2_, *E*_*j*=10_, and Δ*E*) were compared with those of recently reported NiFe-LDH/carbon composite catalysts and related noble-metal-free transition-metal oxide catalysts (Table 7).

**Table 7:** Comparison of bifunctional oxygen electrocatalytic performance (*E*_1/2_, *E*_*j*=10_, and Δ*E*) of the Fe–N–C@NiFe-LDH composite with recently reported noble-metal-free catalysts in alkaline media

Catalyst	Year	*E*_1/2_ [V *vs.* RHE]	*E*_*j*=10_ [V *vs.* RHE]	Δ*E* [V]	Ref.
Fe–N–C@NiFe-LDH	2026	0.849	1.61	0.76	This work
MOF-derived Fe–N–C@NiFe-LDH	2026	0.83	1.68	0.85	49
S–Co_3_O_4_	2025	0.788	1.55^a^	0.76^b^	50
NiFe-LDH-A-Fe/NC-CNT	2024	0.84	1.59	0.74	51
NiFe-LDH/NBC	2025	0.85	1.47	0.62	52
NiFe–NiFe_2_O_4_@NC	2025	0.87	1.55^a^	0.68	53

a *E*_*j*=10_ was converted from the OER overpotential (*η*_10_) reported in the literature according to *E*_*j*=10_ = 1.23 + *η*_10_.

b Δ*E* was calculated from the reported *E*_1/2_ and the converted *E*_*j*=10_. Because the electrolyte concentration, catalyst loading, rotation rate, and *iR*-correction conditions differ among the reported studies, the numerical comparison should be regarded as approximate.

The overall potential gap, Δ*E*, of 0.76 V achieved by the Fe–N–C@NiFe-LDH composite was smaller than that reported for MOF-derived Fe–N–C@NiFe-LDH (0.85 V),^49^ and was comparable to that of S–Co_3_O_4_ (0.76 V).^50^ In contrast, Δ*E* the value obtained in this study was slightly higher than those reported for NiFe-LDH-A-Fe/NC-CNT (0.74 V),^51^ NiFe-LDH/NBC (0.62 V),^52^ and NiFe–NiFe_2_O_4_@NC (0.68 V, calculated from the reported values).^53^ It should be noted, however, that direct numerical comparison of these values across different studies is subject to variation in electrochemical testing parameters, including electrolyte concentration, catalyst loading, rotation rate, and *iR*-correction procedures. Therefore, these comparisons serve primarily as a quantitative benchmark to assess relative performance trends.

Considering the individual oxygen electrode reactions, the ORR half-wave potential (*E*_1/2_) of the present catalyst (0.849 V *vs.* RHE) was comparable to the high values reported for NiFe-LDH-A-Fe/NC-CNT (0.84 V *vs.* RHE)^51^ and NiFe-LDH/NBC (0.85 V *vs.* RHE).^52^ This high level of ORR capability is fully consistent with our XPS and RRDE analyses, confirming that the high-density Fe–N_*x*_ catalytic environments inherited from the SPP-synthesized Fe–N–C substrate—along with a highly selective four-electron (4e^−^) reduction pathway—were successfully retained within the composite structure.

For the OER, the potential required to reach 10 mA cm^−2^ (*E*_*j*=10_) for the present Fe–N–C@NiFe-LDH catalyst was 1.61 V *vs.* RHE, which was lower than that of MOF-derived Fe–N–C@NiFe-LDH (1.68 V *vs.* RHE),^49^ indicating that a lower potential was required to achieve a current density of 10 mA cm^−2^. However, this value remained higher than those reported for NiFe-LDH/NBC (1.47 V *vs.* RHE)^52^ and NiFe–NiFe_2_O_4_@NC (1.55 V *vs.* RHE, converted from the reported overpotential).^53^ These comparative insights indicate that further reducing the OER overpotential represents the primary lever for minimizing the overall potential gap (Δ*E*) in this specific composite architecture.

Overall, the Fe–N–C@NiFe-LDH composite developed in this study exhibits bifunctional oxygen electrocatalytic performance within the range reported for recent noble-metal-free catalysts, while further improvement in OER activity and durability remains necessary. To bridge the remaining performance gap with top-performing reported literature, future optimization strategies should focus on three primary structural targets:

1. Morphological regulation: refining synthesis parameters to reduce the thickness and improve the dispersion and crystallinity of the NiFe-LDH phase, thereby exposing a higher density of active edge/surface Ni/Fe sites.

2. Interface & active site exposure: mitigating the excessive physical coverage of underlying Fe–N_*x*_ centers by stacked LDH nanosheets (as observed in SEM), ensuring unhindered reactant (O_2_/OH^−^) mass transport.

3. Interfacial electronic environment & robustness: optimizing the local electronic environment and improving the mechanical and chemical stability of the Fe–N–C/NiFe-LDH interface are important targets for further improvement. The XPS shift indicates an interfacial electronic perturbation but does not directly establish accelerated electron-transfer kinetics.

## 4. Conclusions

In this study, solution-plasma-derived Fe–N–C was combined with NiFe-LDH through nitric-acid surface functionalization and hydrothermal growth. Acid treatment increased the amount of oxygen-containing surface groups but simultaneously decreased surface N and Fe–N_*x*_ species, shifting the ORR half-wave potential (*E*_1/2_) from 0.894 to 0.852 V *vs.* RHE. Subsequent NiFe-LDH growth yielded a Fe–N–C@NiFe-LDH composite with *E*_1/2_ = 0.849 V, *E*_*j*=10_ = 1.61 V, and Δ*E* = 0.76 V. Notably, the additional decrease in *E*_1/2_ after LDH growth was only 3 mV relative to Fe–N–C_acid, indicating that most of the observed ORR activity loss occurred during the acid-treatment step under the present synthesis conditions.

Durability measurements revealed remaining limitations of the composite. Fe–N–C@NiFe-LDH retained 81.7% of its ORR current after 10 000 s, whereas its OER *E*_*j*=10_ shifted positively by 95 mV after 1000 potential cycles. SEM/EDS, XRD, and XPS support the formation of NiFe-LDH on the Fe–N–C-based support and a change in the local electronic environment upon composite formation; however, the present data do not directly establish the direction or rate of interfacial charge transfer. Further improvement therefore requires milder surface functionalization, controlled NiFe-LDH coverage, and enhanced interfacial durability.

## Author contributions

Conceptualization and methodology, T. I.; experimental and data analysis, K. O., K. Y., H. T., and T. I.; writing – original draft preparation, K. O.; writing – review and editing, T. I.; supervision, T. I.; project administration and funding acquisition, T. I. All authors have read and agreed to the published version of the manuscript.

## Conflicts of interest

There are no conflicts to declare.

## Supplementary Material

RA-016-D6RA07919A-s001

## Data Availability

The data supporting this article are included as part of the supplementary information (SI), which contains the methods for estimating the apparent electrochemically active surface area (ECSA) and evaluating H_2_O_2_ yield and electron transfer number (*n*), Raman spectra and corresponding Raman parameters, H_2_O_2_ yields and electron transfer numbers derived from RRDE measurements, CV curves used to determine the double-layer capacitance and apparent ECSA, and ECSA-normalized LSV polarization curves for the oxygen reduction reaction (ORR) and oxygen evolution reaction (OER) in 0.1 M KOH. Supplementary information is available. See DOI: https://doi.org/10.1039/d6ra07919a.
