# Supplementary material for: Solution-plasma-derived Fe–N–C/NiFe-LDH composite for bifunctional oxygen electrocatalysis: effects of surface modification and LDH growth
Source: RSC Adv. 2026 Oct 16;16(47):63351–67. doi: 10.1039/d6ra07919a (PMC13673662; doi:10.1039/d6ra07919a)
Supplement: Supplementary file 1 [file RA-016-D6RA07919A-s001.pdf]

Supplementary Information (SI) for RSC Advances.
This journal is © The Royal Society of Chemistry

Supporting Information

Solution-plasma-derived Fe–N–C/NiFe-LDH composite for bifunctional oxygen electrocatalysis: effects of surface modification and LDH growth

Kentaro Ogihara[1]，Kouki Yamamoto[1]，Hayato Takada[1], Takahiro Ishizaki[2,*]

[1]Materials Science and Engineering, Graduate School of Engineering and Science, Shibaura Institute of Technology, Toyosu, Koto-Ku, Tokyo, 135-8548, Japan

[2]College of Engineering, Shibaura Institute of Technology, Toyosu, Koto-Ku, Tokyo, 135-8548, Japan

*Corresponding author: ishizaki@shibaura-it.ac.jp

S1. Evaluation of H_2O_2 Yield and Electron Transfer Number

The H_2O_2 yield and electron transfer number (n) were calculated from the disk current (I_D) and ring current (I_R) obtained from RRDE measurements using the collection efficiency (N = 0.37) of the Pt-ring/glassy-carbon-disk electrode according to the following equations:

$$H_2O_2 \text{ yield } [\%] = \frac{200\left(\frac{I_R}{N}\right)}{I_D + \frac{I_R}{N}} \quad (1)$$

$$n = \frac{4I_D}{I_D + \frac{I_R}{N}} \quad (2)$$

where the absolute values of the currents were used for both I_D and I_R. The H_2O_2 yield and electron transfer number (n) as a function of potential are shown in Fig. S2, and the representative values are summarized in Table S2.

S2. Evaluation of Electric Double-Layer Capacitance and ECSA

To estimate the apparent electrochemically active surface area (ECSA) of Fe–N–C, the acid-treated sample, and the composite, cyclic voltammetry (CV) measurements were performed in 0.1 M KOH over a potential range of 1.165–1.260 V vs. RHE at scan rates of 10, 20, 40, 60, 80, and 100 mV s^{-1}. The ECSA was calculated according to Equation (3) using a specific capacitance (C_s) of 40 $\mu F\ cm^{-2}$, as reported in the literature.[43]

$$\text{ECSA} = \frac{C_{dl}}{C_s} \times (A_{geo}) \quad (3)$$

Table S1. Summary of Raman spectroscopy parameters, including the intensity ratios of D-to-G bands (I_D/I_G) and 2D-to-G bands (I_{2D}/I_G), for Fe–N–C, Fe–N–C_acid, and Fe–N–C@NiFe-LDH.

Sample	I_D/I_G	I_{2D}/I_G
Fe-N-C	1.13 ± 0.04	0.17 ± 0.09
Fe-N-C_acid	1.12 ± 0.03	0.14 ± 0.05
Fe-N-C@NiFe-LDH	0.99 ± 0.03	0.32 ± 0.05

Table S2. Calculated electron transfer numbers (*n*) and H_2O_2 yields (%) derived from RRDE measurements for Fe–N–C, Fe–N–C_acid, and Fe–N–C@NiFe-LDH across the potential range of 0.20–0.80 V vs. RHE.

Sample	H_2O_2 yield [%]	Electron transfer number [*n*]
Fe-N-C	< 0.01	4.00
Fe-N-C_acid	2.46	3.95
Fe-N-C@NiFe-LDH	2.54	3.95

Table S3. Apparent ECSA values estimated from C_{dl} for Fe–N–C, Fe–N–C_acid, and Fe–N–C@NiFe-LDH from non-Faradaic CV measurements.

Sample	C_{dl} [mF/cm²]	ECSA [cm²]
Fe-N-C	4.63	14.5
Fe-N-C_acid	3.56	11.2
Fe-N-C@NiFe-LDH	2.46	7.72

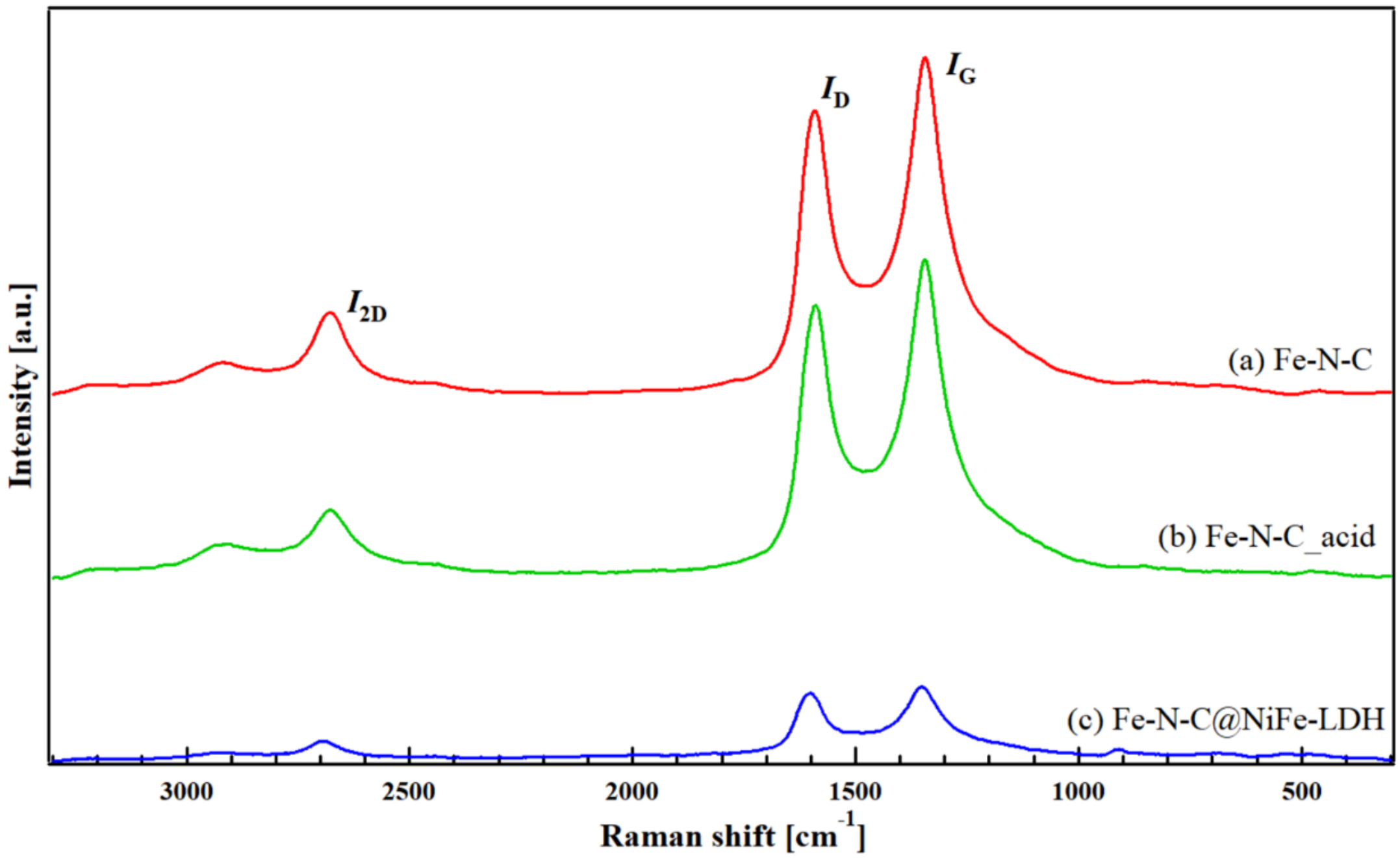

Fig. S1 ．Raman spectra of (a) Fe–N–C, (b) Fe–N–C_acid, and (c) Fe–N–C@NiFe-LDH, displaying the characteristic D, G, and 2D bands used to assess structural defect density (I_D/I_G) and surface optical scattering environment changes.

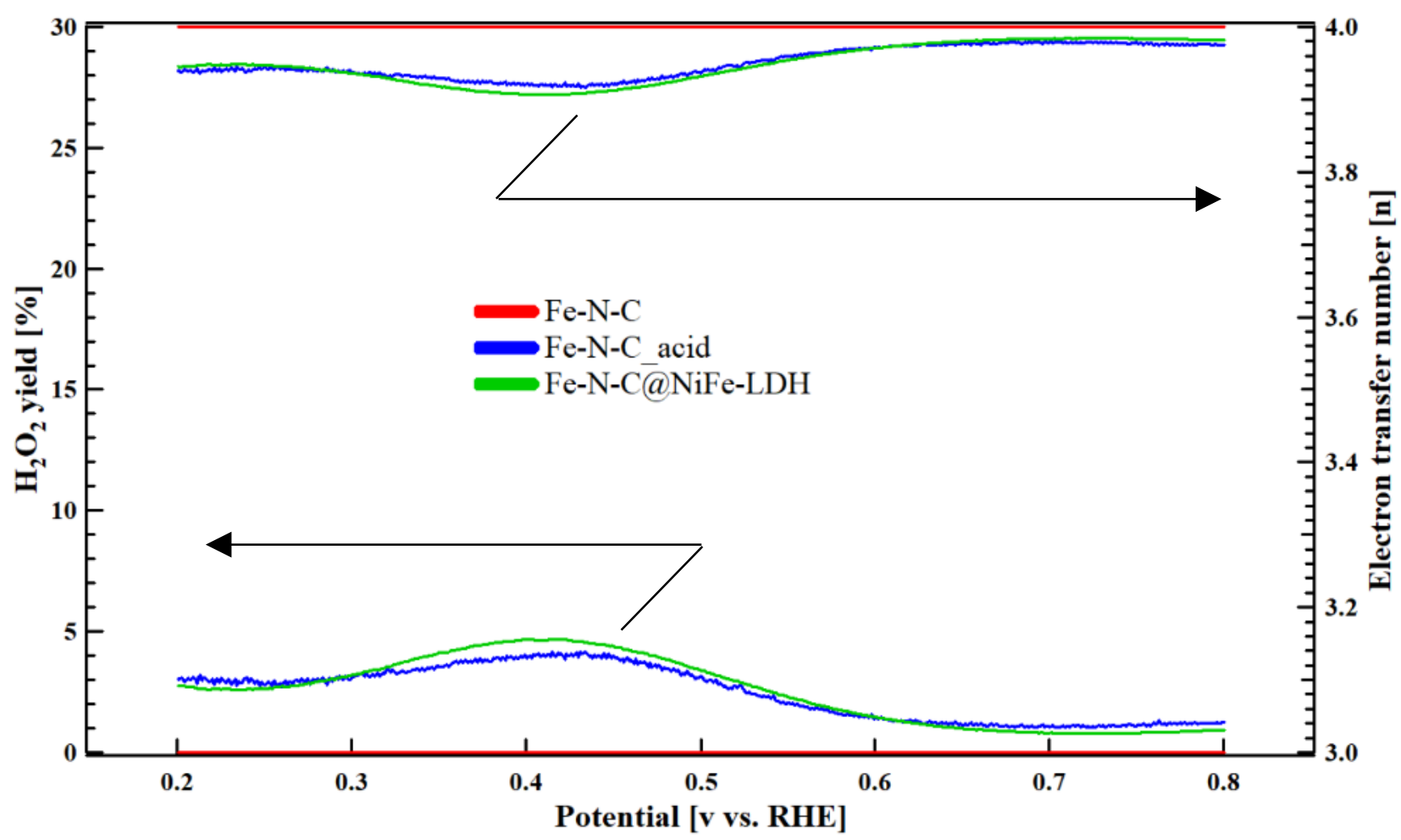

Fig. S2 H_2O_2 yields and calculated electron transfer numbers (*n*) of Fe–N–C, Fe–N–C_acid, and Fe–N–C@NiFe-LDH derived from rotating ring-disk electrode (RRDE) measurements in O_2-saturated 0.1 M KOH.

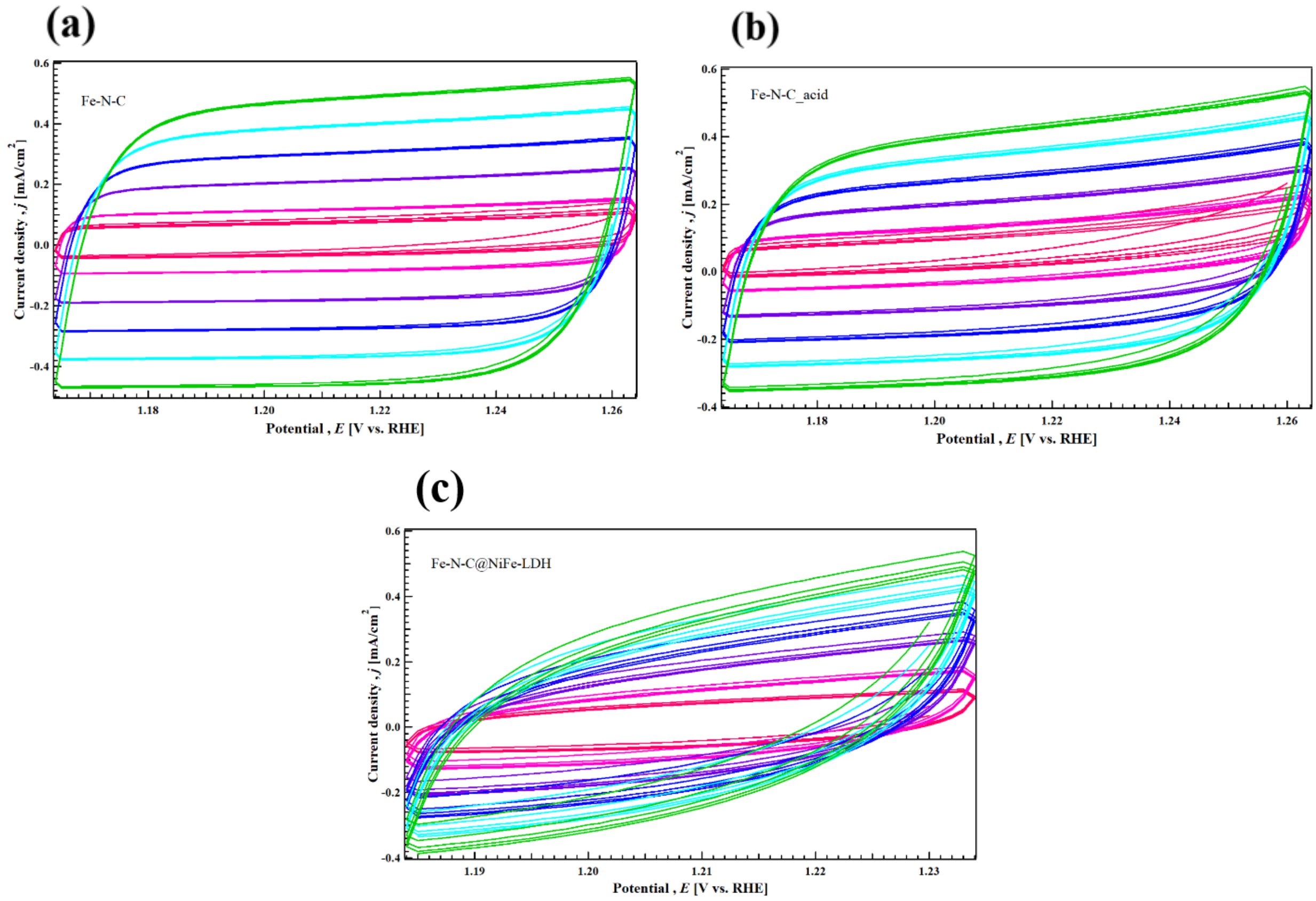

Fig. S3 Cyclic voltammetry (CV) curves recorded in the non-Faradaic potential region at various scan rates (10 to 100 mV s^{-1}) for (a) Fe–N–C, (b) Fe–N–C_acid, and (c) Fe–N–C@NiFe-LDH to determine double-layer capacitance (C_{dl}) and estimate the apparent electrochemically active surface area (ECSA).

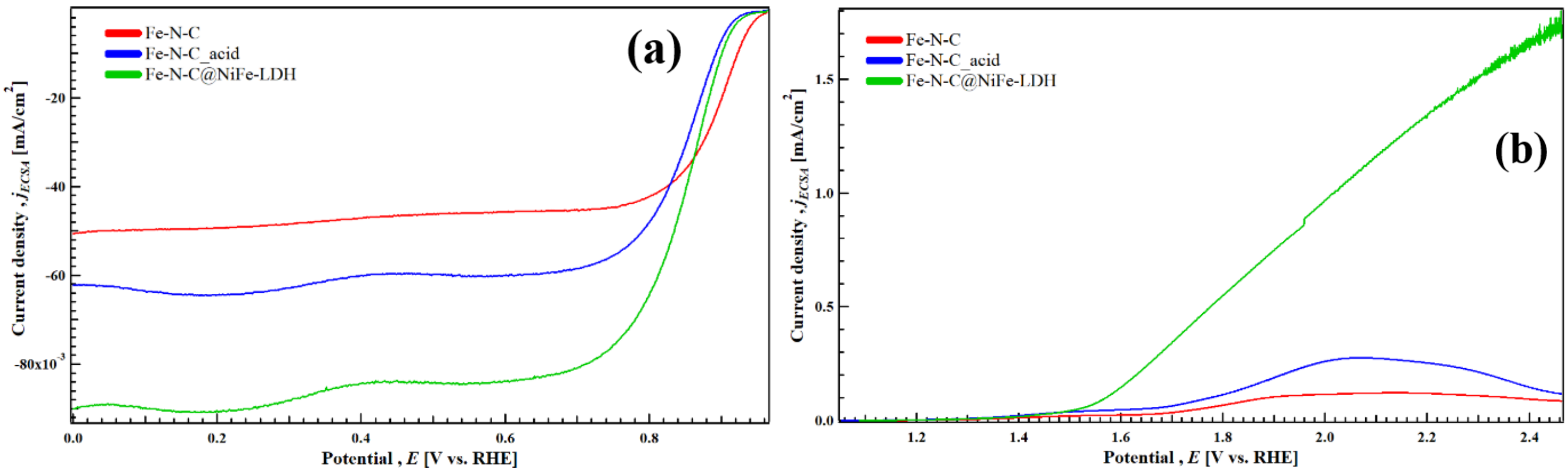

Fig. S4. LSV polarization curves normalized by the apparent ECSA of Fe–N–C, Fe–N–C_acid, and Fe–N–C@NiFe-LDH for (a) ORR and (b) OER. The normalized currents are used for relative comparison of surface-area-normalized activity among the samples.
